# Topic identification, selection, and prioritization for health technology assessment in selected countries: a mixed study design

**DOI:** 10.1186/s12962-024-00513-8

**Published:** 2024-02-06

**Authors:** Julia Bidonde, Vigdis Lauvrak, Aparna Ananthakrishnan, Pritaporn Kingkaew, Elizabeth F. Peacocke

**Affiliations:** 1 Division of Health Services, Norwegian Institute of Public Health, Skøyen, Postbox 222, 0213 Oslo, Norway; 2 School of Rehabilitation Science, University of Saskatchewan, Saskatoon, Canada; 3 Evidence and Health Technology Assessment, EviHTA, Oslo, Norway; 4 Health Intervention and Technology Assessment Program, Ministry of Public Health, Nonthaburi, Thailand

**Keywords:** Technology assessment, Surveys and questionnaires, Priority setting, TISP, HTA, Topic identification, Topic selection, Topic prioritization

## Abstract

**Background:**

There is limited evidence-informed guidance on TISP processes for countries where health technology assessment (HTA) is in a nascent phase. We aimed to explore the range of topic identification, selection and prioritization (TISP) processes and practices for HTA in selected countries and identify aspects relevant to emerging HTA systems.

**Methods:**

This mixed design study included a systematic literature review, an electronic survey, and individual interviews. We conducted a systematic literature review with criteria that were developed a priori to identify countries deemed to have a recently formalized HTA system. Based on the literature review, a twenty-three item online survey was shared with the identified countries, we completed follow-up interviews with ten participants who have experience with HTA. We analyzed documents, survey responses and interview transcripts thematically to identify lessons related to TISP processes and practices.

**Results:**

The literature review identified 29 nine candidate countries as having a “potential” recently formalized HTA system. Twenty-one survey responses were analyzed and supplemented with ten individual interviews. We found variation in countries’ approaches to TISP — particularly between pharmaceutical and non-pharmaceutical interventions. Results indicate that TISP is heavily driven by policy makers, expert involvement, and to a lesser extent, relevant stakeholders. The use of horizon-scanning and early warning systems is uncommon. Interviewee participants provided further insight to the survey data, reporting that political awareness and an institutional framework were important to support TISP. TISP can be optimized by stronger national regulations and legislative structures, in addition to education and advocacy about HTA among politicians and decision-makers. In some settings regional networks have been useful, particularly in the development of TISP guidelines and methodologies. Additionally, the technical capacity to conduct TISP, and access to relevant local data were factors limiting TISP in national settings. Increased network collaboration and capacity building were reported as future needs.

**Conclusions:**

This study provides current insights into a topic where there is limited published peer reviewed literature. TISP is an important first step of HTA, and topics should be selected and prioritized based on local need and relevance. The limited capacity for TISP in settings where HTA is emerging may be supported by local and international collaboration to increase capacity and knowledge. To succeed, both TISP and HTA need to be embedded within national health care priority setting and decision-making. More in-depth understanding of where countries are situtated in formalizing the TISP process may help others to overcome factors that facilitate or hinder progress.

**Supplementary Information:**

The online version contains supplementary material available at 10.1186/s12962-024-00513-8.

## Background

Many countries are adopting Health Technology Assessment (HTA) to make evidence-informed decisions about health technologies and interventions, and to support universal health coverage (UHC) [1, 2]. HTA is a multidisciplinary process using “explicit methods to determine the value of a health technology at different points in its lifecycle” [3]. Topic identification, selection, and prioritization (TISP) is the first step of the HTA process.

The EUR-ASSESS project (1997) conceptualized and suggested considerations for HTA topic prioritization [4]. Soon thereafter several established agencies (mainly in high-income countries) provided information online regarding their TISP processes, including criteria and stakeholder involvement [5, 6]. A recent review of the International Network of Agencies for Health Technology Assessment (INAHTA) member agencies’ approaches to topic selection indicated that most well-formalized agencies follow a six-step process: (i) Specification of criteria for topic selection, (ii) Identification of topics, (iii) Shortlisting of potential topics, (iv) Scoping of potential topics, (v) Scoring and ranking of potential topics, and (vi) Deliberation and decision-making on final topics for HTA [5].

For policy makers in resource-limited countries, where HTA is relatively new or where there may not be the necessary supporting institutional mechanisms, or resources, there is limited evidence-informed guidance on the optimal implementation of TISP processes [7]. HTA often reflects different factors within a country, for example, data usage culture, capacities, priorities and organizational arrangements. Consideration of political, economic, social, and cultural factors can be important for the development of evidence-informed processes and could be relevant when deciding what type of TISP approach a country could adopt [8, 9]. HTA is a resource intensive process; and a transparent and rigorous process for the identification, selection and prioritization of topics can result in better utilization of an HTA agencies’ resources [10, 11]. However, standardized approaches to TISP can be underutilized in some countries, potentially undermining the legitimacy of HTA decisions. Additionally, adopting complex TISP approaches may not be a suitable strategy for all HTA systems. Current guidance for countries establishing an HTA program suggests a pragmatic process at the beginning [12], rather than a more intentional approach to TISP, which can minimize its relevance within the program.

Given the importance that the nomination and selection of topics for assessment has as the first step of an HTA process [10, 13], there is value in investigating and describing best practices and lessons that may be transferrable to a variety of HTA systems. We hypothesized that there are common TISP elements across countries, and a focus on TISP processes can provide important insights into how a country’s HTA system is meeting the health sector’s needs. Therefore, the study's objective was to explore the range of TISP processes and practices for HTA in selected countries and identify aspects relevant to emerging HTA systems.

## Methods

This study comprised a systematic literature review, a survey, and interviews. For the purposes of this study, we defined *a formalized HTA system* as being one where HTA is set up at the national or regional level to work in a predefined manner, with transparent process steps, and with a clear commission to support decisions applicable to the access, financing, and coverage of health services. In contrast, we defined an *emerging HTA system* to describe a setting that’s in the early stages of developing and implementing HTA processes and infrastructure. The term "emerging" implies that the system is actively working towards establishing a functional and comprehensive HTA system, but may still be in the process of developing policies and guidelines, building capacity, and conducting assessments.

### Framework for the study

We developed a framework to interpret and analyze the data, to guide the formulation of survey questions, and to facilitate the integration of existing knowledge into the study. The framework was influenced by the EuroScan Toolkit, as reported by the European network for HTA (EUnetHTA) [7, 14], and further developed using studies identified from the systematic literature review, combined with the team’s expertise. The framework is not prescriptive, and it focus on common elements that are likely to apply in all countries regardless of the stage of HTA institutionalization within the country. It has three components: Topic identification, Topic selection, and Topic prioritization (Table 1).

**Table 1:** Detailed TISP framework (adapted from [15])

Framework component	Definition	Examples
Topic identification	Suitable topics are identified for assessment This stage has three categories: • Reactive (awaiting input from someone) • Proactive (actively searching for topics as part of the HTA system’s mandate or work program) • A mix of proactive and reactive approaches	Examples of proactive approaches include contacting manufacturers, using literature searches and disinvestment strategies. Horizon scanning and early awareness typically is based on a mixture of approaches [14, 46, 47]. Although horizon scanning has been advocated to support collaborative HTA in Europe [7], several countries with experience in HTA such as Thailand [26], most countries in Latin America [25], and many in Europe [48] use simplified approaches to identifying topics
Topic selection (or filtration)	Identified topics are checked for alignment with the aims of the HTA system	For example, if the HTA system has a narrow scope such as childhood vaccination, then vaccines not suitable for children will be excluded. Selection processes may involve technical advice from clinical experts and industry [7, 27]
Topic Prioritization	A decision is made to initiate, reject, or postpone an assessment While selection ensures that identified topics are aligned with the aims of the HTA system, prioritization is needed when not all identified and selected topics may be assessed at the same depth or through the same process due to limited available resources. Ideally, the purpose is to ensure that topics of greatest impact (to the health system), are adequately assessed in time. Prioritization may follow an explicit or implicit ranking process	Standard prioritization criteria are commonly based on predefined criteria for example, disease burden, availability of other treatment, cost, clinical impact. Criteria like the Pritec tool [20] are used by some HTA agencies. Other agencies may have implicit ranking processes

### Systematic literature review

The systematic literature review was conducted to identify and describe TISP options and practices to inform the cross-sectional survey questions, and to identify countries in resource-limited settings (Low and middle income (LMIC)), with a *formalized HTA system* (as defined above) which we could invite to participate in the survey. Full details of the systematic review methods can be found in the project plan and protocol (Additional file 1: S1), and are reported in a previous publication [15]. We also searched PubMed and Scopus (Additional file 2: S2) (multiple searches were conducted between 6 October 2020 and 14 April 2021) and HTA websites (Additional file 1: S1 and Additional file 2: S2).

### Survey

We developed a 23-question survey to identify what processes and practices formalized HTA systems use for TISP (Table 2, Additional file S3), as defined above (Additional file 1: S1). The survey used closed, single or multiple select nominal questions. Many questions included an ‘other’ option for respondents to describe their answer in their own words and provide open-ended responses.

**Table 2:** Measurement outcomes and related survey questions

Measurement outcome	Question
• Expertise question	Consent Do you consider yourself to have the necessary experience and understanding of the HTA system in the country to respond to questions about TISP processes?
1. Background of the HTA system	1 1.1 Please indicate if you agree to the following statement: Your country has a formalized* HTA system to support its population's access to health services 1.2 What type of technologies or interventions are in scope for your country's HTA system(s)? 1.3 On average, how many assessments are initiated by the HTA system(s) in your country each year? 1.4 On average, how many times in one year are topics prioritized for HTA (number of decisions to initiate HTAs) in your country? 1.5 Who is the formal decision maker of the HTA system(s) e.g., who decides on acting or implementing recommendations (nationally or regionally)?
2. How TISP is performed?	2 2.1 How are technologies for HTA identified in your HTA system(s)? 2.2 Does your HTA system(s) use a formalized selection and prioritization process? 2.3 If yes, is selection and prioritization performed using explicit criteria and/or a ranking system(s)? 2.4 Who is involved in the prioritization processes? 2.5 Add any comments on the selection and prioritization process 2.6 What are the outcomes of the TISP process? 2.7 Are outcomes of the TISP process (as listed in the previous question) publicly available? 2.8 Is information on selected, but not prioritized, topics publicly available? 2.9 Any comments about transparency?
3. Factors that have influenced the current TISP processes	3 3.1 What are the main factors that have influenced the choice of TISP process? 3.2 Has the TISP process been influenced by international or regional networks and collaborations? 3.3 If yes, please describe the main source(s) of influence 3.4 Describe attempts that your country/institution has made to improve the TISP processes
4. Future needs	4 4.1 What are the main limitations of your country’s topic identification, selection and prioritization process? 4.2 In your opinion, what would facilitate a more transparent and sustainable process for TISP? 4.3 What do you consider to be the most important technologies or interventions that should be in focus for a future collaborative initiative on the TISP process in your country? 4.4 If there is a need for capacity development in your country, please select those options that apply 4.5 Please add any further comments that you believe are relevant

The target population for both the survey and follow up interviews were individuals who were familiar with their national HTA system. We approached at least one person who we assumed to be familiar with the HTA system, identified through the authors’ network of contacts or contact information on scientific publications. Familiarity with the context was confirmed by the question “Do you consider yourself to have the necessary experience and understanding of the HTA system in the country to respond to questions about TISP processes.” The survey is reported according to the Checklist for Reporting of Survey Studies (CROSS) [16] (Additional file 4: S4).

#### Survey data collection

The survey was piloted by colleagues who had not been involved in its development. The questionnaire took 20–30 min to complete and was hosted on the online SurveyMonkey platform (Momentive Inc software company, United States) (www.surveymonkey.com). All operational definitions (e.g., formalized HTA system) were included in the survey (Additional file 3: S3).

In April 2021, we sent email invitations to 48 individuals associated with 29 countries (Table 3, Additional file 5: S5, Additional file 6: S6) with three follow-up emails to non-responders. Invitees were asked to forward the email to the appropriate person within their organizations if they did not have knowledge in specific areas. No incentives were offered for participation in the survey.

**Table 3:** Countries invited to participate and response in survey and follow up interview

N	Formalized HTA system identified in literature review	Income level of country (classified by WB 2021)	No. invited to survey	No. participated in survey	No. invited to follow up interview	No. of follow up interviews
	**Africa**					
1	South Africa	UM	3	1	2	2
	**Asia Pacific**					
2	Korea, Rep	H	1	NR	1	NR
3	Malaysia	UM	1	1	1	1
4	Singapore	H	2	NR	2	Declined
5	Taiwan, China	H	1	NR	1	NR
6	Thailand	UM	1	1	1	1
	**Europe**					
7	Bulgaria	UM	1	Unqualified	1	NR
8	Croatia	H	4	1	2	NR
9	Czech Republic	H	1	1	1	NR
10	Estonia	H	2	1	2	1
11	Hungary	H	3	1	2	Declined
12	Kazakhstan	UM	1	1	1	1
13	Latvia	H	3	1	3	NR to written follow up
14	Lithuania	H	2	1	3	Agreed after deadline
15	Poland	H	1	2	1	NR
16	Romania	H	4	1		NR
17	Serbia	UM	2	1	2	1
18	Slovak Republic	H	2	1	2	1
19	Türkiye	UM	1	1	1	NR
20	Ukraine	LM	1	Incomplete	–	–
	**Latin America**					
21	Argentina	UM	3	1	1	NR
22	Brazil	UM	2	NR	1	NR
23	Chile	H	4	1	2	1
24	Colombia	UM	2	1	2	1
25	Mexico	UM	2	NR	0	-
26	Peru	UM	2	NR	1	NR
27	Uruguay	H	1	NR	1	NR
	**North Africa & Middle East**					
28	Iran, Islamic Rep	LM	1	1	1	NR
29	Tunisia	LM	1	1	1	NR
	Total		55	21	39	10

*LM* Lower middle-income country, *UM* Upper middle-income country, *H* High-income country, *NR* No response, Unqualified: did not have competence to complete; Incomplete: skipped every question; Declined: replied to email declining participation

#### Survey analysis

We prepared simple descriptive statistics using the quantitative survey data and analyzed the written survey responses thematically, looking for lessons related to TISP processes and practices. Responses were anonymized and reviewed for clarity and completeness. The initial results were presented to survey respondents during a virtual workshop June 2021, the discussion at this meeting was incorporated into the results.

#### Follow-up interviews

To gain further insight into the survey results we conducted follow-up interviews during April–May 2023. We sent a single email invitation with a summary of the survey results to 27 countries (39 individuals) asking them to join a follow-up meeting (Ukraine was not invited to interview due to the Russian invasion, and we had not secured a respondent from Mexico in the survey, so they too were excluded). Four either declined, did not provide a written response, or agreed after the deadline. Fourteen did not respond (Table 3). Reminder emails were sent to four individuals with the aim of including follow-up interviews from all regions included in the survey. Individuals who accepted the invitation were sent an email with the summary of results and the interview guide (Additional file 7: S7, Additional file S8). JB and EP conducted ten interviews (nine on Microsoft Teams and one on Zoom) in English using open-ended questions. Interviews were 36–58 min in length and participants consented to interviews being recorded. The recordings were transcribed, and thematic analysis was performed by one author (JB) and audited by a second (EP). No incentives were offered to the interviewees.

## Results

### Systematic literature review results

The literature search retrieved 1094 records. After title and abstract screening, we classified 294 records by region (Africa, Asia, Latin America, and Europe) and country and included 64 records with some information on the status of an HTA system. The only LMICs that met our definition of a *formalized HTA system* were the Islamic Republic of Iran, Tunisia, and Ukraine. To align with our aim to explore the range of TISP processes and practices, therefore, we expanded the list of eligible countries to include Upper middle-income countries, or those with recently *formalized HTA systems* (e.g., former members of the Union of Soviet Socialist Republics), as we anticipated that they could have more transferable experience relevant to emerging HTA systems. Twenty-nine candidate countries were identified as having a “potential” recently formalized HTA system. One was from Africa, five from Asia Pacific, 14 from Europe, seven from Latin America, and two from the North Africa & Middle East region (Table 3). For some reports, the information was not detailed enough for the authors to judge whether the HTA system was formalized (Additional file 6: S6).

### Survey and interview results

**Fig. 1:**
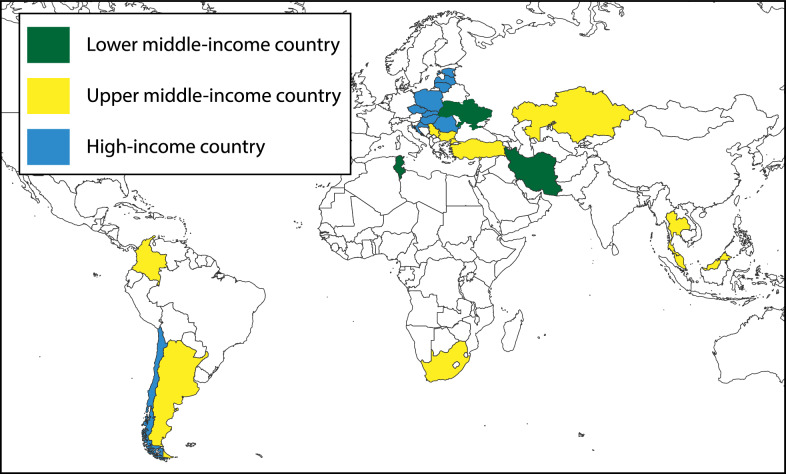
Geographical distribution of survey respondents

Three of the 29 countries identified for the survey were categorized as middle income, three were lower middle income, 12 were upper middle income, and 14 were high income (according to the 2021 World Bank development indicators [17] (Fig. 1, Table 3).

#### Respondent characteristics

We received 23 survey responses from 21 countries (response rate 72%). One respondent indicated that they did not have experience and understanding of the HTA system in their country and discontinued the survey. Due to emailing several people from the same country, we received four responses from two countries, one respondent requested have their submission removed at the end of the survey. For the other country (that also had two responses), the responses are reported individually as there is no way to verify if one response was more accurate than the other. An additional three respondents were unsure if their country had a formalized HTA system, however we included their responses in the results because the country had been identified as eligible from our literature review (Table 3, Additional file 3: S3).

#### Respondents for the follow-up interviews

We recruited ten individuals for the follow-up interviews. The participants were from Africa (n = 2), Asia Pacific (n = 2), Europe (n = 4), and Latin America (n = 2).

#### HTA system background information

**Fig. 2:**
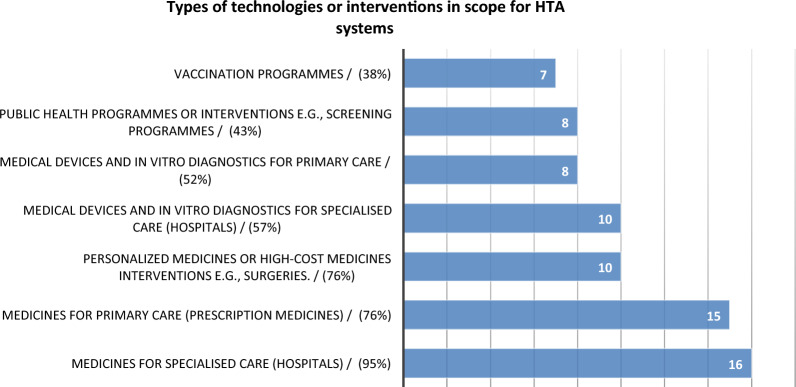
Types of technologies or interventions in scope for HTA systems

Figure 2 shows the reported types of technologies assessed in the HTA systems and Table 4 shows the number of assessments and the prioritization processes. The scope of considered technologies or interventions encompassed specialized care medicines^1^ (n = 20 countries), primary care medicines (n = 16), high-cost care medicines^2^ (n = 16), medical devices (n = 11), public health programs (n = 9), and vaccination programs (n = 8). Interviewees noted that the number of technologies included in an agency’s scope depended on several factors, with resources being a significant consideration:

- *“So there are other technologies which are not assessed at all. Uh, because of the limited capacity...”*

**Table 4:** Survey results: average number of assessments and topics prioritized by the HTA system(s) each year

Number of assessments initiated each year					Number of topics prioritized each year				
Number	Pharmaceutical interventions		Non-pharmaceutical interventions		Number	Pharmaceutical interventions		Non-pharmaceutical interventions	
	No	%	No	%		No	%	No	%
1–10	5	25	5	25	1–2	10	59	10	71
11–20	2	10	4	20	3–6	1	6	1	7
21–50	6	30	4	20	> 6	4	24	3	21
51–99	3	15	–	0	Do not know	2	12	–	–
100 +	3	15	–	0	Number of. responses	17		14	
Do not know	1	5	1	5					
Number of responses	20		20						

The largest volume of pharmaceutical assessments was between twenty-one and fifty products per year (n = 6). Volume was lower for non-pharmaceuticals (range: 0–50 per year). Six respondents said their agency did not initiate non-pharmaceutical assessments and five respondents reported that they did not know. As one participant explained, the ability to complete non-pharmaceutical evaluations may be limited to lack of data. In some cases pharmaceuticals and non-pharmaceutical interventions are processed by different agencies or the choice is influenced by available finances where for example, there is less funding and demand for HTAs related to non-pharmaceutical topics. Our study found that whether the HTA body receives a pharmaceutical dossier from industry can be an influential factor in a country’s approach to TISP. For example, the Republic of Slovakia has the maximum threshold value for assessing the cost-effectiveness of medicines cost-effectiveness threshold explicitly stated in legislation [18, 19], and this means that all medicines under the maximum threshold value are selected as topics for HTA without a prioritisation process.

Survey respondents identified formal decision makers of the HTA systems, including those implementing recommendations, as: (1) policy makers, (2) payers and insurance agencies, and (3) committees. Interviewees reported a lack of awareness and understanding among decision-makers regarding the complexity and nuances of the HTA decision-making process which, in turn, hinders effective and transparent outcomes. Political influence exacerbates these challenges, potentially compromising objectivity, and integrity in decision-making. A lack of knowledge about HTA and evidence-based medicine, and the political nature of decision-making are issues identified that hinder successful HTA and TISP:

- *“Nobody knows how it is and how it should be …even the Ministry*”
- “*There's a lack of understanding in terms of what [HTA] it is and what it's meant to do.”*
- *“Even in the Ministry. People are not … aware. People even are not aware of [name] law [and] how it is functioning. So, …everything is, you know, functioning due to inertia.”*

An interviewee who described their country’s HTA processes as performed by more than one body reflected on the value of one decision-maker within an HTA system, stating *“If you are going to use HTA as a tool for decision-making, then you need to understand that you can't have decision-making pockets everywhere.”* Another interviewee describes decision-makers’ lack of awareness and understanding saying:

- *“In my opinion, if a country spends some money for health technology assessment, it needs to improve the decision-making process because if they don’t, then it’s a waste of money.”*

When decision-making lacks transparency, survey and interviewees’ responses reported that this compromised the public trust and integrity of the system, potentially leading to dissatisfaction and challenges to the legitimacy of the decisions.

Topic proposals were most typically made by a government department, government officials, or HTA system decision-makers (n = sixteen) (see Table 5). Interviewees remarked that: *“also the Ministry of Social Affairs has suggested some topics”* another one added *“actually the Ministry just recently suggested a topic”.*

- *“[identification happens] via negotiations with [the] Ministry of Health, regarding the HTA topics, including the initial topics proposed by the Ministry”.*

**Table 5:** Survey responses: How topics are identified

	Number of responses (n = 20)	% (of responses)
Proposed by a government department or government officials	16	80
Proposed by health care workers/experts	13	65
Proposed by manufacturers	13	65
Proposed by the HTA systems decision maker	13	65
A formalized topic identification process	11	55
Proposed by patients or the public (including academics/citizens)	9	45
Proposed by those producing the HTA evidence	8	40
Proposed by those receiving the HTA report	7	35
A formalized early warning system/horizon scanning process	5	25
Other reason given	3	15

*HTA* health technology assessment

For the decisions about topic identification participants reported that these decisions were commonly made by Ministries of Health, National Health Services or the Ministry of Research, an interviewee said:

- *There is huge problem, completely uncovered…orphan drugs and rare diseases…orphan drugs are paid in our country since [year] and that achievement was political…the decision from the Ministry of Health was made to pay those drugs.*

Some respondents highlighted inconsistency between centralized and regional processes. The terms ‘payers’ or ‘health insurance agencies’ were both used (under the stewardship of Ministries of Health), indicating the impact of differences in health system design on HTA and TISP. The types of committees involved in HTA could be a national board for benefits packages, a drug committee for medicines and vaccines, and HTA committees who reported directly to the Ministry of Health.

#### How TISP is performed

Survey results for topic identification were heterogeneous. Proactive approaches (horizon scanning or early warning systems) were uncommon (n = five). Eleven (55%) respondents indicated that their TISP was reactive. Interviewees commented: “*… in [region name] parts of Europe we are doing more reactive approach”* which they associated to a few things one of them being part of a “small market.”

- *“...they [decision-makers] do not see the necessity of investing in scanning activities, and for me it is mind boggling because it is more of a reactive system than a proactive system, right?”*

Almost all respondents (n = 18, mean = 4.75, median = 5) reported that more than one stakeholder group was involved in topic identification. In some cases topics are also proposed by health care workers or experts (n = 13) and industry (i.e. manufacturers of drugs/devices) (n = 13). Interviewees commented:

- “*what is working, or at least on paper, is of course identifying the topics and the classification … It happens through a committee … this committee is looking at the [World Health Organization] Essential Medicine List and through, you know, any new data that is coming in.”*
- “*most of the topics come from the health insurance fund as they have had some more complex applications for drugs services”.*

On the use of explicit criteria for topic selection and prioritization and the use of ranking systems, survey responses were mixed. Nine respondents (50%) reported that prioritization was performed using explicit processes and criteria. Seven respondents (39%) provided the criteria used in their country, which included estimates of the burden of disease, potential impact on costs, clinical and organizational effects and domestic ethical, equity or mandated considerations as required by national legislation. Interviewees said:

- “*We have [an] official criteria for prioritization. Mainly impact, morbidity, mortality, economic burden, clinical tools used in clinical practice sometimes. The criteria are decided by our Ministry of Health, but the process of prioritization includes also the [centre’s name].”*
- *“the country has a process to prioritize conditions and treatments called "[name]” in health. This process is enshrined in the law and includes a package of [number] prioritized conditions for treatment and financial protection.”*

One respondent reported using the Pritec tool [20]. Two respondents provided links to publicly available criteria [21, 22]. Three respondents reported that they used criteria, but did not elaborate further and another noted that criteria were being piloted. One interviewee suggested topic selection and prioritization may be done on an ad hoc basis by committees appointed without a clear process. Comments included:

- *“We don’t have a formal prioritization process. We don’t have any formal process yet for that [prioritization].”*
- *“They [the committee] will say, let's see, which of these drugs should be prioritized… And they have, based on their experience and their “own needs”* [emphasis added by authors].

#### Transparency and the availability of the outcome of TISP decisions

Information on the availability and transparency of TISP outcomes is mixed. Ten respondents (59%) said that TISP outcomes (e.g., a list of topics, alerts, vignettes or short notes, and reports of relevant technologies within specified areas) were not publicly available, and eleven (65%) reported that information on the topics selected was also not publicly available. Seven respondents (41%) stated that neither the outcomes nor the selected topics were publicly available, meaning that in these countries there is no public information on the identification of topics. The survey free text responses shed some light on the process, indicating that information could be made available “on reasonable request,” or it was available for selected groups. Interviewees said:*“…it could be more transparent, but I would say that for the regional standards is transparent. “*

- *“I think [TISP] it's relatively transparent… [the] health insurance fund, is a public organization, so decisions have to be very transparent…the meeting notes are available to the public, so it's possible to understand how the decisions were made and also when we kind of put together the reports.”*

Others suggested that the current system was not transparent and some mentioned that their organization was currently working on improving transparency. An interviewee said *“…the decision-making process is opaque and not well-described.”* In some cases, the independent agency responsible for HTA assessment was not responsible for or able to influence transparency in other parts of the HTA system, or its related processes (i.e., the Ministry of Health has sole authority):

- “*I feel the [TISP] process … could be more transparent, but this part of the process is not doesn't depend on the [agency] it depends on the Minister”.*
- *“it's mostly problem of the decision-making process because this process is unclear and fragmented”*

Interviewees noted that stakeholders can influence transparency. Patients’ groups may exert significant influence on the HTA process by voicing patients’ needs and preferences, or pharmaceutical manufacturers may exercise influence:

- “*[there are] some vocal groups involved putting themselves on the agenda… [the process is] “hijacked” by some groups”.* [emphasis added by authors].
- “*But in [year], pharma industry said that we are too transparent and so if we are too transparent that they will not deliver drugs to [country’s] reimbursement system because all other countries will see the prices and it can create problems for their businesses in other countries”.*

#### Factors influencing the current TISP processes

The survey indicated several factors relevant to the type of HTA TISP process (Table 6). The process is reported to be limited to policy makers and expert involvement (n = 11). Interviewees agreed that “*TISP is a political decision”.* Other factors that influenced a country’s choice of TISP were its participatory nature and the involvement of all or most relevant stakeholders (n = eight), or politics and context (i.e., a political decision) (n = six). Some interviewees referred to the appointment of experts by the government for TISP, inferring that they may not be politically neutral.

**Table 6:** The main factors that have influenced the choice of TISP process

	n. (of 17 responses)	% (of responses)
A process limited to policy makers and expert involvement	11	65
A participatory process involving all or most relevant stakeholders	8	47
A political decision	6	35
Other reason given	4	24
Don’t know (unmet needs, demand for coverage, limited HTA research capacity, and political pressure)	1	6

Three non-response countries reported that there are no TISP processes and the topics are proposed by manufacturers or the manufacturer and decision-maker (by submission or by request)

Study participants highlighted the influence of international and regional networks’ published guidance and collaborations on TISP processes, including those published by OSTEBA, INAHTA, HTAi, NICE, EVIDEM, and EUnetHTA. Eight participants (47%) reported that these networks had a significant impact. Networking activities occurred at international and local levels, with international agencies playing a crucial role offering guidance on methods and facilitating mentoring opportunities:

- *“We feel and we need to participate [at] different [periods of time] and with different association[s], like HTA International (HTAi) or INAHTA and for example in the Americas we’re working with the RedETSA (the HTA network f the Americas) and EVIDEM at different points and because we use the methods.”*

The EUnetHTA core model was a topic of discussion in several interviews:

- *“…participation within the European Network for Health Technology Assessment (EUnetHTA JA2 and EUnetHTA JA3 projects) have significantly improved the quality of the process of HTA in [country].”*

For countries with emerging HTA systems, survey and interview results indicate that those that collaborate with international partners have built capacity, although achieving sufficient levels of education and expertise around HTA continues to be challenge and will take time. Our study’s results provided insights into the links between HTA bodies and countries’ broader education systems, it appears that some interviewees saw the value in establishing relationships and collaborations with local universities. The integration of health and education systems seemed to be essential enabling factors for producing HTAs. Engaging with universities is a factor influencing the TISP process, in terms of supporting capacity building efforts, knowledge innovation, development, dissemination, and in some cases developing the HTA reports. Leveraging universities’ expertise and resources enhances the TISP process by fostering collaboration, facilitating skill development, and promoting the exchange of valuable knowledge within the local innovation ecosystem:

- “*The HTA Center in [country] is under the University of [name].”*
- *“Increas[ing] capacity is very important. We need the local educational program. Maybe some master programs…this is very important for us, we have to work on capacities”*

The interviews identified other important factors influencing the TISP process. Firstly, the upcoming implementation of a new European regulation [23] that will come into effect in 2025, and is anticipated to change TISP and HTA processes within Europe. Secondly, misalignment between procurement and selection timelines was identified as a challenge, leading to inefficiencies and decision-making delays. Interviewees noted the issue of wasteful duplication of HTA efforts by different agencies, sometimes even within the same country, highlighting the need for better coordination and collaboration among stakeholders. Interviewees’ impression of the lack of awareness and understanding of HTA by decision-makers meant that some participants felt it was important to educate Ministry of Health staff and clinicians about the information required for TISP and assessments, since their understanding and involvement are crucial in driving informed decision-making. Despite widespread recommendation, participants reported limited utilization of HTA in the decision-making process, indicating a gap between recommendations and policy implementation.

#### Factors limiting TISP and future needs

Survey and interview respondents described a range of current issues that require attention, including the lack of topic selection processes, weak regulatory or legislative processes, decisions being irrelevant for policy makers, or incomplete TISP criteria. Some of these issues relate to the HTA system in general, such as the fragmentation of HTA as a decision-making tool in the health sector. Participants widely reported inconsistency between the use of HTA in some contexts. Human resource capacity was flagged many times as a limitation for TISP, and the HTA system more generally. HTA agencies with limited finances may struggle to train and develop their staff, and also experience the migration of skilled workers to health technology and pharmaceutical companies because of higher salaries and enhanced career prospects that industry offers. This loss of expertise poses a significant challenge to the effective functioning of HTA agencies, particularly in resource-limited countries:

- *“I don‘t want to lose any people on my team… to educate them and give the training is very - very complex. It's a long - long process and the people of the pharmacy wants to come here to [the] institute and take the people with the double or three, three times more money.”*

Eleven respondents (52%) noted attempts to improve the TISP process within their country, centered around either adjustments to the types of criteria or the revision or weighting of criteria. Other efforts related to strengthening the general HTA processes, such as improving process transparency through publishing information on websites, initiating meetings with stakeholders and international partners, awareness raising and training and capacity building.

Results indicate that HTA agencies need resources, with examples of the duplication of HTA efforts, by public or private entities, suggested to be unproductive. Interviewees reported challenges around data availability and adaptation to local contexts, and certain technologies could lack proper assessment as a result.

The lack of patient and public participation is another factor influencing the TISP process, and identified as a future need in survey and interview. Interviewees agreed there is no established procedure for patient or community involvement in TISP, although some reported seeking input from interest groups, or at the later steps of the HTA process (e.g., analysis, appraisal, or decision-making). They suggested that patient groups can submit proposals to the Ministry of Health or the HTA body, but limited capacity hinders a more active or engaged involvement. Often patient groups are not part of the TISP (or HTA) committees, with healthcare professionals dominating decision-making. One participant said, “…*involvement of indigenous populations and communities is important but not adequately addressed.”* Overall, efforts are being made to improve patient and public engagement, but further work is required:

- *“We have an understanding of the necessity to involve patients and our citizens in decision-making process, but sometimes our decision-making process is a little closed”.*
- *“Uh patient and citizen. It’s well known, you know that the government is, is struggling with that even though you will see in [place] that there is a lot of activity and interventions happening at the community level and it’s so important involve communities.”*

#### TISP insights for countries with emerging HTA systems

Interviewees acknowledged the complexity of offering a universally applicable list of suggestions and emphasized that countries with an emerging HTA system can benefit from other countries experiences. Drawing from the survey and interviews, a set of interconnected key messages were identified:

- **Governance**: some participants reported it is important to establish an autonomous and independent agency for HTA that is insulated from political influences. Participants believed the institutional framework is key setting the direction and mechanisms that will support TISP and other HTA decisions being based on scientific evidence and objective analysis rather than political considerations. Critical aspects of governance include transparency and accountability built into the TISP process and the presence of an enabling environment for collaboration and adequate resourcing.
- **Political awareness and support**: The awareness, support and understanding of policy makers is another important consideration that was identified through interviews. Participants suggested that information cannot trigger actions if it is not well understood. Education and advocacy about all steps of HTA to politicians and decision-makers can support TISP, and HTA and overall stewardship of health systems.
- **Effective coordination**: TISP requires a multi-stakeholder process: an emphasis on fostering a positive culture of collaboration, with clearly defined roles and responsibilities of the various actors involved plus clear lines of accountability is also crucial.
- **Guidelines and Methodologies**: although there is currently no universal consensus among countries on this, it would be important to establish agreed guidelines and methodologies for conducting TISP.
- **Regional networks**: developing partners can play a critical role helping establish and build TISP/HTA. Regional and international initiatives such as EUnetHTA or RedETSA, seem to have been a useful for sharing best practice, and there may be value in further investment and engagement in more regional networks.
- **Technical Capacity**: TISP and HTA processes depend in large part on the availability of a broad range of specialized skills. These processes are inherently multidisciplinary and require the involvement of a variety of disciplines. Technical capacity determines many features that influence TISP. The technical ability of staff can facilitate or inhibit the process. Setting unrealistic expectations of what a HTA body can deliver (due to ineffective TISP), may lead to inadequate coverage and undermine the credibility of the HTA process. A high turnover may weaken the overall technical capacity which should mitigated by strategizing on how retaining current staff.
- **Identification in Data-Scarce Scenarios**: selecting the right topic for an HTA depends on accessing data, for example, the issue of access to epidemiological data is necessary to understand the priority of the problem was highlighted. Further, the quality of HTA and their usefulness for decision making is directly linked to the quality and completeness of data. When data are lacking, conducting field research and engaging with patient organizations and academia can provide insights into emerging health technology needs and topics of concern.

## Discussion

This study aimed to understand how TISP is performed in different countries through identifying factors that influence a country’s choice of TISP, particularly those relevant to countries with emerging HTA systems. The need to establish priorities for which technologies are to be assessed is a long-standing important issue for the HTA community [4, 24]. We found variation in TISP approaches, similar to previous findings [5, 25–27] with TISP being very closed in some counties (e.g., limited to policy makers and expert involvement), with the potential for being much more inclusive and participatory (e.g., involving all or most relevant stakeholders). At a strategic level, political awareness and governance supported through institutional frameworks were raised as important to TISP. It was suggested by several participants that regional networks have been a useful tool in some settings, particularly in the development of guidelines and methodologies. Factors relevant at a country level included the technical capacity to conduct TISP, and access to relevant local data.

Some survey respondents and interviewees noted that there were both real and perceived political influences in HTA decisions. Stakeholder pressure has been identified as a factor that may influence an HTA body’s priority towards “hot topics” [28], that can sometimes come from people in positions of power. Chinitz (2004) discusses the role of HTA in health policy making, exploring the increased politicalization of HTA. It is suggested that originally these assessments occurred in relatively depoliticized environments where assessments were “politically innocuous” studies of technologies that were considered to be of high importance, although over time these decisions have evolved into more high-profile decisions [29]. Culture, values and institutional context may influence the use of HTA [30], with political buy-in identified as a barrier for HTA in general, and a supportive political environment being relevant for its implementation [31, 32]. The responses from our study suggest that political influence continues to be a factor in the selection of topics, as well as in down-stream HTA decisions and implementation of the technology.

In this study, TISP was characterized as having relatively low patient or public involvement (Table 5). As interviewees mentioned, this may reflect the need for additional resources to recruit and support the involvement of patient and public groups. It may also suggest issues of historical-political trust issues between government institutions and citizens. Patient or public involvement in HTA is reported as increases the credibility and transparency of reimbursement decisions [33], which could also be relevant in the TISP process. A well-recognized example of participatory approaches in health governance is Thailand’s National Health Assembly that was designed to secure greater public participation and consultation in health policy making and has been credited with successfully enabling more meaningful public engagement in health policy decisions [34]. Other examples from participatory budgeting literature highlight the effectiveness of stakeholder engagement in decision-making include Brazil, Italy, and India [35, 36]. A gap that was acknowledged by many interviewees in their TISP process was the recognition that public awareness-raising activities and formal feedback processes should be put in place, or developed further, to increase the engagement of the patient and citizen groups in health policy decisions. However, it was also acknowledged by interviewees that it takes time, and is reported in other studies that it places greater demands on capacity and coordination skills to ensure the necessary representatives are engaged in the process [34].

Transparency is key for a fair priority setting processes in all steps of HTA, including TISP [37]. Transparency considerations include which stakeholders are involved in TISP, documenting the basis of decisions, publishing the decisions, and describing options for appealing decisions [38–42]. The interviews offered insight related to the transparency of TISP decisions. Some interviewees discussed a very open process, where all industry dossiers were reviewed (and publicly available), however other interviewees reported that decisions about topics for HTA were made behind closed doors, or by the Minister. Transparency can have strengths and weaknesses for an HTA system. On the positive side, a transparent process lays bare the decision-making process, and additionally may support the real-world applicability of the HTA recommendations. A transparent process encourages decision-makers to be more systematic and explicit, and by default, publicly accountable [43]. On the negative side, for the system to be very transparent, it requires time and resources to share the information, and as a result may delay the process. The information itself may also be overly complex for the public at large. And there may be instances where secrecy is necessary for legitimate reasons e.g., as a requirement by manufacturers.

Despite countries working to improve their TISP processes by including more explicit criteria and making final decisions more accessible and publicly available, the study suggests that there continues to be challenges with the TISP methodology. Qiu et al. suggested that consensus approach for the development of methods of topic selection would be valuable for the HTA community [44]. However, as TISP needs adapt to different contexts, it is difficult to develop standard methods guidance. Despite this, having explicit criteria and making information on decisions available in the public domain should support better TISP processes, particularly in countries that have several prioritization processes and HTA programs. Many suggestions made during this research on improving TISP processes indicated the need to consider the overarching HTA system, including the need to provide adequate human and financial resources. Success with TISP, and HTA in general, seems to rely on embedding it within the national health care decision-making and priority setting system [45]. HTA can be supported through legislation to regulate and implement the various stages of the HTA process. The more governments and other stakeholders appreciate the usefulness of HTA the greater the likelihood that HTA production process will be sufficiently resourced. Increased public engagement with (and ownership of) the TISP process within both HTA and the decisions arising from it may generate more enthusiasm and demand for such priority-setting processes.

Finally, establishing a TISP mechanism within an emerging HTA system is a process, but not necessarily a linear process, nor a binary one; one cannot say if a country has a full TISP process or not. Instead, it seems is a dynamic, complex and ongoing process with many influences, often happening in parallel involving multiple players, stakeholders, continues learning and changes.

## Strengths and limitations of the study

The strengths of this study are that the responses provide a current and unique insight into TISP processes in a wide range of countries, particularly relevant to emerging HTA systems. The responses outline factors to consider when planning to implement HTA in support of UHC. There is little published information about TISP processes for countries where HTA is in a nascent phase, and often the processes are not reported in detail, so this information from actors within HTA systems is particularly valuable. However, despite the relatively high response rate (72%) to our survey, it was necessary to follow up with individual interviews for additional detailed responses. We cannot guarantee that survey respondents and interviewees were the most knowledgeable people on TISP processes within their organizations. Our findings are indicative and require further in-depth research.

## Conclusion

This study provides current information on the range of processes and practices influencing TISP in countries with a recently formalized HTA systems. Despite the attention paid to HTA as a catalyst in achieving UHC, TISP in many countries is still developing. TISP is not conducted in any standard manner, lacks transparency in some countries and varies in terms of the degree of collaboration achieved with stakeholders, including the public. Additionally, there are challenges driven by a lack of awareness of the HTA process, few experts and adequate resources for the system were mentioned by many of the individuals in our sample. International and local collaborative approaches to TISP and capacity building were valued by respondents and initiatives such as EUnetHTA, REDESTA, and the EuroScan international toolkit for early awareness. Our findings suggest that TISP reflects national cultures and practices, influenced by each county’s implicit governance structures. Broadly, despite TISP requiring consideration of individual country contexts, including the political setting and available resources, a comprehensive and consistent approach to TISP can support the HTA system.

### Supplementary Information

**Additional file 1: S1.** Project plan: Topic identification and Selection for Health Technology Assessment (HTA), options for Low- and Middle- Income Countries (LMICs).

**Additional file 2: S2.** Search strategy.

**Additional file 3: S3.** Survey.

**Additional file 4: S4.** CROSS Survey reporting checklist.

**Additional file 5: S5.** Countries per region meeting the inclusion criteria.

**Additional file 6: S6.** Included country list by region.

**Additional file 7: S7.** Summary of findings.

**Additional file 8: S8.** Interview guide.

## Data Availability

The datasets used and/or analyzed during the current study are available from the corresponding author on reasonable request.

## Abbreviations

EUnetHTA: European network of Health Technology Assessment
EVIDEM collaboration: Evidence and Value Impact on Decision Making collaboration
HITAP: Health Intervention and Technology Assessment Program
HTA: Health Technology Assessment
HTAi: Health Technology Assessment International
INAHTA: International Network of Agencies for Health Technology Assessment
LMIC: Low-and middle-income country
NICE International: National Institute of Care and Health Excellence International
NIPH: Norwegian Institute of Public Health
OSTEBA: Office of Basque Health Technology Assessment
TISP: Topic identification, selection and prioritization
UHC: Universal Health Coverage

## Footnotes

1 Specialized care medicines: medications specifically designed and tailored for the treatment of specific medical conditions or patient populations, at the specialist level of care (often hospital level).

2 High-cost medicines: significant financial burden due to their high cost.
