# Supplementary material for: Topic identification, selection, and prioritization for health technology assessment in selected countries: a mixed study design
Source: Cost Eff Resour Alloc. 2024 Feb 6;22:12. doi: 10.1186/s12962-024-00513-8 (PMC10848436; doi:10.1186/s12962-024-00513-8)
Supplement: Supplementary file 1 — Additional file 1: S1. Project plan: Topic identification and Selection for Health Technology Assessment (HTA), options for Low- and Middle- Income Countries (LMICs). [file 12962_2024_513_MOESM1_ESM.pdf]

Topic identification and Selection for Health Technology Assessment (HTA), options for Low and Middle Income Countries (LMICs)

Project plan for delivery 3.2b, Topic identification and selection in low and middle income countries (LMICs), Work package 2, Comprehensive Approach Towards Universal Health Coverage.

Summary

Health technology assessment (HTA) is widely recommended as a means to support universal health coverage (UHC). The first step of an HTA process may be referred to topic identification, selection and prioritisation (TISP). Our aim is to describe options for TISP relevant for low and middle income countries (LMICs). The project will be conducted in three steps. First we will perform a scoping search for relevant literature on TISP tools, how TISP is set up in LMICs with an established HTA system, and collaborative initatives. Then, we will approach LMICs with established HTA systems through a survey to explore what factors and needs have influenced their choice of a particular TISP process. In the third step we will transcribe, interprete and discuss our findings and suggest options for TISP with particular relevance for LMICs. The main endproduct of the project will be a report with the most relevant identified options with pros and cons for each option described. The report will be published and used to support capacity building for HTA in LMICs.

Title:

Topic identification and Selection for Health Technology Assessment (HTA), options for Low and Middle Income Countries

----

Project plan for delivery 3.2b, Topic identification and selection in low and middle income countries (LMICs), Work package 2, Comprehensive Approach Towards Universal Health Coverage.

Commissioned by:

Funded by NORAD, commissioned internally.

Start date:

01.12.2019.

End date:

01.06.2021

Project Team:

Vigdis Lauvrak, Senior advisor, project leader, NIPH
Elizabeth Peacocke, Senior advisor, NIPH
Julia Bidonde, Senior researcher, NIPH

Internal peer review:

Frønsdal Katrine, Senior researcher, NIPH

Lumbwe Chola, Senior researcher, NIPH

Approved by:

Ingvil Sæterdal, Department Director, NIPH, Global Health

Mandate

This is a plan for a project on how the first step of an HTA process, Topic identification, selection and prioritisation (TISP), can be shaped to be relevant and sustainable in low and middle income countries (LMICs). The project deliverables represents delivery 3.2b, TISP in LMICs, Work package 2 of the project Comprehensive Approach towards Universal Health Coverage (CA-UHC). The aim is to prepare a report with options on the TISP step relevant for LMICs. The project is proposed and coordinated by the Norwegian Institute of Public Health, and financed by the Norwegian Agency for Development Cooperation (NORAD) for the period 2018-2021.

Background

The CA-UHC project

The Comprehensive Approach towards Universal health Coverage (CA-UHC) project is led by The National Institute of Public Health in Norway and funded by the Norwegian Agency for Development Cooperation (NORAD) running from 2018 to 2021 (1). The CA-UHC project has three strategic objectives:

- Improved evidence base to set priorities at national level in pursuit of UHC.
- Effective tools and digital solutions assisting countries in achieving UHC.
- Improved global knowledge to advance current thinking and support progressive realisation of UHC.

The objectives of the CA-UHC are covered by four work packages (WPs):

- WP1 A New Lancet commission
- WP2 Scale up of Health Technology Assessment (HTA)
- WP3 Systematic Reviews for Health Systems Strengthening in LMICs
- WP4 Building concerted e-healt solutions for quality care, monitoring and benchmarking of UHC in maternal and child health

This project plan describes sub-delivery 3.2b of WP 2 concerning means to identify and select topics for HTA.

HTA

HTA[1] is an internationally accepted multidisciplinary approach to analyse and assess evidence to inform health policy (2). HTA is widely recommended as a means to support UHC (3, 4). HTA informed decision processes, also referred to as HTA systems or HTA frameworks, can be described through generic process steps as shown in Figure 1.

[1] HTA is a multidisciplinary process that uses explicit methods to determine the value of a health technology at different points in its lifecycle. The purpose is to inform decision-making in order to promote an equitable, efficient, and high-quality health system (2).

Figure 1. Generic steps of decision-making pathways involving HTA

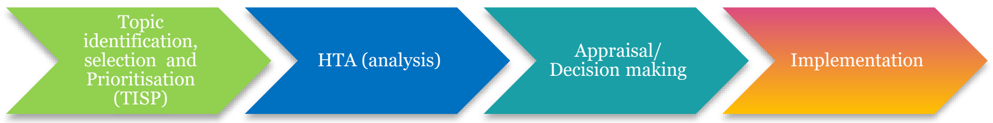

Some HTA systems focus only on new pharmaceuticals, while other systems have a broader focus, including any technologies to be covered by a compulsory insurance fund or other means of public funding. The scope of a HTA system will strongly influence how the first step of the HTA process, i.e. the TISP step (described in the next section) is set up. Relatively few LMICs have established structured HTA systems (4-6), but those that have, may provide information that can be translated to options and tools for other LMICs to consider.

Identification, selection and prioritisation of topics for HTA

Identification and selection is the step were topics are identified and selected according to scope of the HTA system. A prioritisation step is needed if not all identified topics set by the scope can be assessed. A TISP step involving horizon scanning[2] has been described in several recent publications (6, 7) and horizon scanning is recommended for TISP in European cooperation on HTA (8). Based on these publications, horizon scanning is considered to be the most comprehensive tool to be used for unbiased and transparent TISP processes (6, 8). However, as only a limited number of countries having an HTA system actually use horizon scanning to inform initiation of HTA (6, 9), other options for the TISP step might also be relevant for LMICs. There has been performed mappings of HTA systems in LMICs (10-12), but we are not aware of in depth investigations related to the TISP step and why a particular option or model for this step is chosen.

Technologies still considered new in LMICs might be implemented or even considered to be obsolete by health care services in High Income Countries (HICs). This makes it uncertain whether sharing of topics or horizon scanning reports from HICs is of same value in identifying and selecting topics for HTA in LMICs. Based on the above, we consider that options for TISP to be identified in this project should not be restricted to horizon scanning alone, and not be restricted to methods of sharing topics from HICs HTA systems.

[2] In the context of health technology assessment (HTA), Horizon Scanning is the systematic identification of health technologies that are new, emerging or becoming obsolete and that have the potential to affect health, health services and/or society.

Importance of the project

To our knowledge, options for TISP in HTA to support UHC in LMICs has not been systematically investigated and reported on so far. If we can identify and describe options for the TISP process as well as factors and needs that may influence the choices of option in LMICs, the results may be used to inform capacity building projects and support policy-makers in LMICs planning to implement HTA to make informed choices.

Aims

We aim to:

1) Perform scoping searches to:
   a) identify methods, tools and collaborative initiatives for TISP in HTA systems
   b) identify LMICs with established HTA systems[3]
2) Develop a survey and approach selected LMICs, who have an established HTA system to explore how TISP is performed, what have influenced their choice of option for TISP and what they consider as their future needs. In particular, we aim to determine aspects that can be generalised to inform policy makers in LMICs planning to implement HTA to support UHC.
3) Provide a report on options for TISP to inform HTA systems in support of UHC in LMICs.

[3] A country with an established HTA system to inform UHC will be defined as a country were HTA is set up on national or regional level to work in a predefined manner, with defined process steps, and with a clear commission to support decisions relatable to UHC

Methods

Scoping searches

We will perform systematic scoping searches for recent (2015 and later) publications describing, respectively:

a) tools, methods and collaborative initiatives relevant for the TISP step of HTA systems in general (both in HIS and LMIC)
b) HTA systems in LMICs

Restriction to publications from 2015 and later are made as a pragmatic choice between completeness, resources available, and the fact that HTA systems and methods change rapidly. Furthermore, we are aware of recent relevant publications that will be included restricting the need to go further back in time. Inclusion criteria are defined by the topic to be studied and outcomes as described below.

Topic to be studied:

We aim to a) identify and describe options and practices of TISP to inform HTA and b) identify candidates for the survey on factors influencing choice TISP step in LMICs.
Outcomes:

- Methods, tools, criteria and collaborative networks/initiatives for TISP steps in general (no limitation to LMICs)
- LMICs with an HTA system to support UHC, and if available:
    - the scope and overall processes of the HTA system
    - how TISP is performed (including who is responsible for decisions, who performes the steps and how HTA agencies units are involved)
    - reported factors influencing the choice of TISP process
    - transparency of the TISP step

Study design:

Systematic reviews, scoping reviews, studies, reports or web-publications relevant to the research questions will be included.

Search strategy and Information sources:

An information specialist at NIPH will develop the search strategies together with the project leader, and another information specialist will review the search strategy.

The literature search will be set up and conducted by an information specialist in at least the following databases: PubMed, Scopus, Global Medicus index, Open Science Framework.

Additional relevant information will be identified by the project-team members through, web-pages and contacts with at least the following organisations/networks:

- Health Technology assessment international (HHTAi, www.htai.org)
- The International Network of Agencies for Health Technology Assessment (INAHTA (http://www.inahta.org/)
- The European Network for Health Technology Assessment (EUnetHTA, www.eunethta.eu)
- The Asia-Pacific research network on HTA (HTAsia link, https://www.htasialink.org/)
- The HTA network of the Americas (RedETSA, www.redetsa.org)
- Euroscan international network (www.euroscan.org)
- The International Horizon Scanning Initiative, (IHSI, https://ihsi-health.org/
- The Profesional Society for Health Economics and Outcome Research (ISPOR (https://www.ispor.org/)
- The International Decision Support Initiative (iDSI, https://idsihealth.org/)
- The World Health Organisation (WHO, https://www.who.int/health-technology-assessment/en/).

Inclusion of literature

First, two project team members will independently go through all identified titles and abstracts and categories these according to if they are publications fulfilling the inclusion criteria defined by the study topic and outcomes. Then, records included based on titles and abstracts will be retrieved in full-text and inspected independently by two project team members. Any conflicting results will be solved by discussions with the project leader.

Records not fulfilling the inclusion criteria defined by topic and outcomes will be excluded

Extraction and presentation of data

Data to be extracted are mainly qualitative data on the predefined outcomes. Standard data extraction templates will be prepared and piloted. Data will be extracted by one team member and checked by another. The data will be presented as narratives of main findings for each predefined outcome.

Survey to selected LMICs

Selection of Countries

LMICs having an established HTA system relatable to UHC will be identified by the scoping search. A system relatable to UHC will be defined as a system where HTA is set up on national or regional level to work in a predefined manner, with defined process steps, and with a clear commission to support decisions relatable to UHC. LMICs with HTA set up to only support local decisions at health institutions, or LMICs were HTA is used only occasionally will not be included. The selected countries will, as far as possible represent LMICs with geographic spreading, different Gross-National Income (GNI), population sizes. Only countries listed by the Organisation for Economic Co-operation and Development that are eligible for Official Development Assistance (ODA) (13) will be selected.

At least one key informant per selected country/HTA system will be sought.

Preparation of the survey

An online survey will be developed and used to approach selected key informants of LMICs who have an established HTA system[4].

To develop the survey, we will recruit at least one key informant from one of the LMICs identified in the scoping search (described above) to provide advice on the survey questions and to pilot the survey.

The survey will include questions related to:

- The HTA system:
  - Scope,capacity and main stakeholders
- The TISP step of the HTA system:
  - The method, transparency, strengths, weaknesses, opportunities, threats, facilitators and barriers for the option chosen
  - Future perspectives for the TISP step
  - Opportunities for (other) LMICs to collaborate

The final wording of the questions will be influenced by the findings of the scoping searches and by the piloting step.

---

[4] A country with an established HTA system to inform UHC will be defined as a country were HTA is set up on national or regional level to work in a predefined manner, with defined process steps, and with a clear commission to support decisions relatable to UHC

Selection of Countries

LMICs having an established HTA system relatable to UHC will be identified by the scoping search. A system relatable to UHC will be defined as a system where HTA is set up on national or regional level to work in a predefined manner, with defined process steps, and with a clear commission to support decisions relatable to UHC. LMICs with HTA set up to only support local decisions at health institutions, or LMICs were HTA is used only occasionally will not be included. The selected countries will, as far as possible represent LMICs with geographic spreading, different Gross-National Income (GNI), population sizes. Only countries listed by the Organisation for Economic Co-operation and Development that are eligible for Official Development Assistance (ODA) (13) will be selected.

At least one key informant per selected country/HTA system will be sought.

Preparing a report to support capacity building

A draft report (Draft report 1) based on the findings from the scoping search and the survey will be prepared and presented at a virtual workshop during spring 2021. Participants of the workshop will include those responding to the survey and collaborative partners of NIPH in the process of setting up HTA systems LMICs. Based on the findings and feedback from the workshop, a report will be produced presenting options for the TISP step (Draft report 2). After peer review the final report will be published and available free of charge on the NIPH web-page. The report is supposed to be used as a support in capacity building related to HTA in LMICs.

Risk of bias

The risk of bias with regard to presenting TISP options will be reduced by conducting the systematic search and to be transparent with regard to both inclusion and exclusion of country examples. Furthermore, we will involve a partner from at least one LMIC to support developing and piloting the survey. The protocol (this document) will be made public available.

Peer –review

The project plan (this document) has been reviewed by all authors and by two peer reviewers internally at NIPH. Internal reviewers at NIPH will also review any publication of results at the NIPH web-site. Additional external peer review will apply to publication of results in scientific journals or other sources (to be determined)

Timeframe, Milestones

Start: 01.05.2020
End: 01.06.2021

Process steps and Milestones	**Start date**	**End date**
Planning and development of Project plan	01.05.2020	15.08.2020
Literature search	01.09.2020	01.10.2020
Inclusion of studies	21.09.2020	12.10.2020
Extraction of data for literature report	12.10.2020	16.11.2020
Finalizing a draft of the literature review	**16.11.2020**	**14.12.2020**
Peer reviewing the draft literature review	14.12.2020	15.02.2021
Developing the draft survey	**15.10.2020**	**15.12.2020**
Inclusion of countries and piloting the survey	15.12.2020	22.01.2021
Performing the survey	22.01.2021	**05.02.2021**
*Summarizing results of the survey (**Draft report 1**)*	05.02.2021	**15.03.2021**
Virtual stakeholder meeting	15.03.2021	25.03.2021
*Preparing a report on options for TISP (**Draft report 2**)*	15.03.2021	**15.04.2021**
Peer-reviewing	15.04.2020	14.05.2021
Preparing the final report ***(Deliverable 1)***	14.05.2021	**01.06.2021***

*The date reflects the delivery of the project report. Publication of results may be influenced by peer review processes

Milestone 1: Draft of literature review finalised
Milestone 2: Draft of survey completed
Milestone 2: Survey performed
Milestone 3: Draft report 1 finalised
Milestone 4: Draft report 2 finalised
Milestone 5: Project report finalised

Deliverables

Results will be published, as:

1. A report with guidance on options for the TISP step to be published on the NIPH web-page.
2. At least one scientific paper based on the literature review and survey results
3. Policy briefs for countries supported in the CA-UHC project (to be determined)

Related projects NIPH

CA-UHC (1)

References

1. Bergh A. Comprehensive Approach to Universal Health Coverage. Oslo, Norway: Norwegian Agency for Development Cooperation 2018-2021.
2. O'Rourke B, Oortwijn W, Schuller T. The new definition of health technology assessment: a milestone in international collaboration. International Journal of Technology Assessment in Health Care. 2020:1-4.
3. (WHO Guidance for institutionalizing HTA mechanisms for reimbursement, unpublished work, 25.06.2020). .
4. WHO. World Health organization, USING HEALTH TECHNOLOGY ASSESSMENT FOR UNIVERSAL HEALTH COVERAGE AND REIMBURSEMENT SYSTEMS Geneva, Switzerland 2-3 November 2015 2015 (https://apps.who.int/medicinedocs/documents/s22250en/s22250en.pdf. Accessed 08.05.2020).
5. Towse A, Devlin, N., Hawe, E., Garrison, L. The Evolution of HTA in Emerging Markets Health Care Systems: Analysis to Support a Policy Response. 2011.
6. Mundy L, Trowman R, Kearney B. Sustainability of healthcare systems in Asia: exploring the roles of horizon scanning and reassessment in the health technology assessment landscape. Int J Technol Assess Health Care. 2020:1-8.
7. Hines P, Hiu Yu L, Guy RH, Brand A, Papaluca-Amati M. Scanning the horizon: a systematic literature review of methodologies. BMJ Open. 2019;9(5):e026764.
8. Lauvrak V, Arentz-Hansen H, Di Bidino R, Erdos J, Garrett Z, Guilhaume C, Migliore A, Scintee SG, Usher C, Willemsen A. Recommendations for Horizon Scanning, Topic Identification, Selection and Prioritisation for European Cooperation on Health Technology Assessment. EUnetHTA WP4 Deliverable 4.10, Oslo, 2020. https://eunethta.eu/services/horizon-scanning/.
9. EUnetHTA. EUnetHTA WP7 Analysis of HTA and reimbursement procedures in EUnetHTA partner countries. 2017; Available from: https://www.eunethta.eu/national-implementation/analysis-hta-reimbursement-procedures-eunethta-partner-countries/ (Accessed February 18 2020).
10. 2015 Global Survey on Health Technology Assessment by National Authorities. Main findings.I.World Health Organization.
ISBN 978 92 4 150974 9 2015.
11. Babigumira JB, Jenny AM, Bartlein R, Stergachis A, Garrison Jr. LP. Health technology assessment in low- and middle-income countries: a landscape assessment. Journal of Pharmaceutical Health Services Research. 2016;7(1):37-42.
12. Tantivess S, Chalkidou K, Tritasavit N, Teerawattananon Y. Health Technology Assessment capacity development in low- and middle-income countries: Experiences from the international units of HITAP and NICE. F1000Research. 2017;6:2119.
13. OECD. Development Assistance Committee List of Official development assistance Recipients: Organisation for Economic Co-operation and Development; 2020 [cited 2020 29 June]. Available from: http://www.oecd.org/dac/financing-sustainable-development/development-finance-standards/daclist.htm.
