# Supplementary material for: Topic identification, selection, and prioritization for health technology assessment in selected countries: a mixed study design
Source: Cost Eff Resour Alloc. 2024 Feb 6;22:12. doi: 10.1186/s12962-024-00513-8 (PMC10848436; doi:10.1186/s12962-024-00513-8)
Supplement: Supplementary file 2 — Additional file 2: S2. Search strategy. [file 12962_2024_513_MOESM2_ESM.docx]

Additional File 2 – Search strategy

The systematic search was performed in PubMed and Scopus. Literature searches were performed on October 6^th^, 2020, and the PubMed search continued until April 14^th^, 2021. The search strategies combined text words for HTA-system, HTA-framework, HTA organizations, and the Cochrane Effective Practice and Organization of Care (EPOC) group filter for Low- and Middle-Income Countries (Available at. <https://epoc.cochrane.org/lmic-filters>), which selects information from countries in Africa, Latin America, Asia and Eastern Europe.

| **PubMed continued until April 14^th^ 2021** |
| --- |
| (((technology assessment, biomedical[mh] OR "technology assessment"[tw] OR "technology assessments"[tw] OR "biotechnology assessment"[tw] OR "biotechnology assessments"[tw] OR HTA[tw] OR HTAs[tw]) AND (agency[tw] OR agencies[tw] OR system[tw] OR systems[tw] OR framework*[tw] OR process*[tw] OR model*[tw] OR policy[tw] OR policies[tw] OR network*[tw] OR organization*[tw] OR organization*[tw] OR OG[sh])) OR"technology assessment, biomedical/OG"[mh]) **AND** [Cochrane Effective Practice and Organisation of Care (EPOC) group filter for Low and Middle Income Countries] **AND** 2015:2021[dp] |
| SCOPUS search performed October 6^th^ 2021 |
| TITLE-ABS-KEY("technology assessment*" OR "biotechnology assessment*" OR HTA OR HTAs) AND TITLE-ABS-KEY(agency OR agencies OR system OR systems OR framework* OR process* OR model* OR policy OR policies OR network* OR organization* OR organization*) **AND** [Cochrane Effective Practice and Organisation of Care (EPOC) group filter for Low and Middle Income Countries] **AND** PUBYEAR AFT 2014 AND NOT INDEX(medline) |

In addition, we searched the following pre-defined websites

- Health Technology assessment international (HTAi, [www.htai.org](http://www.htai.org/));
  The International Network of Agencies for Health Technology Assessment (INAHTA (<http://www.inahta.org/>);
  The Asia-Pacific research network on HTA (HTAsiaLink, <https://www.htasialink.org/>);
  The HTA network of the Americas (RedETSA, www.redetsa.org);
  EuroScan international network (www.euroscan.org);
  The International Horizon Scanning Initiative, (IHSI, <https://ihsi-health.org/>;
  The Professional Society for Health Economics and Outcome Research
  (ISPOR (<https://www.ispor.org/> );
  The International Decision Support Initiative (iDSI, <https://idsihealth.org/>);
  The World Health Organization (WHO, [https://www.who.int/health-**technologyassessment/en/**](https://www.who.int/health-technologyassessment/en/)**).**
