# Supplementary material for: Topic identification, selection, and prioritization for health technology assessment in selected countries: a mixed study design
Source: Cost Eff Resour Alloc. 2024 Feb 6;22:12. doi: 10.1186/s12962-024-00513-8 (PMC10848436; doi:10.1186/s12962-024-00513-8)
Supplement: Supplementary file 3 — Additional file 3: S3. Survey. [file 12962_2024_513_MOESM3_ESM.pdf]

**Welcome to this survey on Topic Identification, Selection and Prioritisation (TISP) processes for Health Technology Assessment (HTA).**

**You have been selected to participate in this survey as your country was identified in our literature review as an African, Asian, Latin American or Eastern European country with a formalised HTA system.**

**The aims of this survey are to:**
**- explore how TISP is performed in selected African, Asian, Latin American and European countries with a formalised HTA system**
**- seek information on what has influenced a country's choice of option for TISP**
**- seek information on what is considered as future needs for TISP in a country's HTA system(s)**

**Completing the survey**
**We are seeking your professional opinion on TISP processes in your country, knowing that this will be your subjective assessment. If you do not feel that you have the expertise to answer the questions, please inform elizabeth.peacocke@fhi.no**

**The survey consists of four sections and must be completed in one sitting (you cannot save your answers and return at a later time).**

- **The HTA system**
- **How TISP is performed**
- **Factors that have influenced the selected TISP process**
- **Future needs**

**Please allocate 30 minutes to complete the survey.**

**Privacy and confidentiality**
**Participation in this survey is voluntary and all information pertaining to respondents will be kept confidential. We will aggregate the data and use it in a report and possibly submit for a scientific publication. Your answers will contribute to disseminate information on TISP processes for low and middle income countries.**

**Dissemination of survey results planned June 2021**

**We will present initial results of the survey in a virtual workshop planned for June 2021. The aim of the workshop will be to offer information on TISP processes for HTA in LMIC. The workshop will be open to all participants approached to complete the survey, and an invitation will follow in due time.**

**We thank you for your time and willingness to respond.**

**If you have any questions, please email: Elizabeth.Peacocke@fhi.no**

**What is the name of the country that your survey response relates to?**

**Do you consider yourself to have the necessary experience and understanding of the HTA system in the country to respond to questions about Topic Identification, Selection and Prioritisation (TISP)* processes?**

**By Topic Identification Selection and Prioritisation (TISP) we mean: The process leading to a topic being identified and prioritised for HTA*

- ◯ Yes
- ◯ No

## 1. The HTA system and how TISP is performed

1.1 **Please indicate if you agree to the following statement**

Your country has a formalised* HTA system to support its populations access to health services.

**A system where HTA is set up at national or regional level to work in a predefined manner, with defined process steps, and with a clear commission to support decisions applicable to the access, financing and coverage of health services (Universal Health Coverage)*

- ◯ Yes
- ◯ No
- ◯ Unsure

1.2 **What type of technologies or interventions are in scope for your country's HTA system(s)?** (check all that apply)

*(NB. There may be different systems depending on the type of technology, respondents are asked to select all types of technology where there is a formalised system).*

- ☐ Medicines for specialised care (hospitals)
- ☐ Medicines for primary care (prescription medicines)
- ☐ Medical devices and in vitro diagnostics for specialised care (hospitals)
- ☐ Medical devices and in vitro diagnostics for primary care
- ☐ Personalized medicines or high-cost medicines interventions e.g., such as surgeries or renal replacement or chemotherapy/ART
- ☐ Public health programmes or interventions e.g., screening programmes
- ☐ Vaccination programme
- ☐ Don’t know

Other, please state

____________________

1.3 **On average, how many assessments are initiated by the HTA system(s) in your country each year?**

| | 1-10 | 11-20 | 21-50 | 51-99 | 100+ | Don't know |
|---|---|---|---|---|---|---|
| Pharmaceutical interventions | ◯ | ◯ | ◯ | ◯ | ◯ | ◯ |
| Non-pharmaceutical interventions | ◯ | ◯ | ◯ | ◯ | ◯ | ◯ |

1.4 **On average, how many times in one year are topics prioritized for HTA (number of decisions to initiate HTAs) in your country?**

| | 1-2 | 3-6 | > 6 times | Don't know |
|---|---|---|---|---|
| Pharmaceutical interventions | ○ | ○ | ○ | ○ |
| Non-pharmaceutical interventions | ○ | ○ | ○ | ○ |

1.5 **Who is the formal decision maker of the HTA system(s) e.g. who decides on acting or implementing recommendations (nationally or regionally)?**

## 2. Topic Identification Selection and Prioritisation (TISP) process (i.e. horizon scanning)

### Topic Identification

Topic identification describes the step where topics for HTA are identified and prioritised.

*By Topic Identification Selection and Prioritisation (TISP) we mean: The process leading to a topic being identified and prioritised for HTA.*

*By Horizon scanning we mean: The systematic identification of health technologies that are new, emerging or becoming obsolete and that have the potential to effect health, health services and/or society. Relying on horizon scanning is one option for a proactive TISP process.*

2.1 **How are technologies for HTA identified in your HTA system(s)?** (check all that apply)

- ☐ Topics may be identified through a formalised early warning system/horizon scanning process
- ☐ Topics may be identified through a formalised topic identification process
- ☐ Topics may be proposed by a government department or government officials
- ☐ Topics may be proposed by health care workers/experts
- ☐ Topics may be proposed by manufacturers
- ☐ Topics may be proposed by those receiving the HTA report
- ☐ Topics are proposed by the HTA systems decision maker
- ☐ Topics may be proposed by those producing the HTA evidence
- ☐ Topics may be proposed by patients or public (including academics/citizens)

Other or comment, please state

________________________________________

### Selection and prioritisation

Topic selection (also referred to as filtration) describes the step were identified or proposed topics are selected based on the scope of the HTA system.

*By Topic prioritisation we mean: the step where the topics identified and selected are ranked or organized. If all selected topics are prioritized, there is no need for a separate prioritisation step.*

2.2 **Does your HTA system(s) use a formalised selection and prioritisation process?**

- ◯ Yes
- ◯ No

2.3 **If yes, is selection and prioritisation performed using explicit criteria and/or a ranking system(s)?**

- ◯ Yes
- ◯ No

If yes, please provide the criteria and ranking systems or indicate if we may contact you for this at a later stage

## Prioritisation

2.4 **Who is involved in the prioritisation processes** (check all that apply)?

- ☐ Employees of an institution (government, non-governmental) that is responsible for Horizon scanning or topic identification
- ☐ Those performing HTA
- ☐ Medical experts appointed by the Government
- ☐ Patients or public (including academics/citizens)
- ☐ Manufacturers
- ☐ Other, please state

2.5 **Add any comments on the selection and prioritisation process**

## Products of the TISP process

### 2.6 What are the outcomes of the TISP process?

- ☐ A list (or lists) of topics
- ☐ Alerts, Vignettes or short notes
- ☐ Reports of relevant technologies within specified areas
- ☐ None of the above

Other (please specify)

2.7 **Are outcomes of the TISP process (as listed in the previous question) publicly available?**

- ◯ Yes
- ◯ No

2.8 **Is information on selected, but not prioritised, topics publicly available?**

- ◯ Yes
- ◯ No

2.9 **Any comments about transparency?**

## 3. What hasinfluenceda country’s choice of option for TISP

3.1 **What are the main factors that have influenced the choice of TISP process** (check allthat apply):

- [ ] A politicaldecision
- [ ] A process limited to policy makers and expert involvement
- [ ] Aparticipatoryprocess involving all or most relevant stakeholders
- [ ] Don’t know
- [ ] Other (please specify)

3.2 **Has theTISP processbeen influenced byinternational or regional networks and collaborations?**

- ○ Yes
- ○ No

3.3 **If yes, pleasedescribethe main source(s) of influence**

3.4 **Describe attempts that your country/institution has made to improvetheTISPprocesses**

## 4. Future needs

4.1 **What are the main limitations of your country's topic identification, selection and prioritization process?**

4.2 **In your opinion, what would facilitate a more transparent and sustainable process for TISP?**

4.3 **What do you consider to be the most importanttechnologies or interventions that should be in focus for a future collaborativeinitiative on the TISP process in your country?** Organize the topics below based on priority (1 most important to 5 least important)

| | 1 | 2 | 3 | 4 | 5 |
|---|---|---|---|---|---|
| Medicines | ○ | ○ | ○ | ○ | ○ |
| Vaccines | ○ | ○ | ○ | ○ | ○ |
| Disease specific HTA (malaria, TB, HIV etc) | ○ | ○ | ○ | ○ | ○ |
| Medical devices and in vitro diagnostics ( | ○ | ○ | ○ | ○ | ○ |
| Public health programmes or interventions e.g., screening programmes, vaccination programmes | ○ | ○ | ○ | ○ | ○ |

Other, please state

4.4 **If there is a need for capacity development in your country, please selectthose options that apply**

- ☐ Political, ministry and legislative level
- ☐ The level of the HTA end-user and decision maker
- ☐ HTA agency or academic level
- ☐ Individual/expert level
- ☐ Patient, public level
- ☐ Manufacturers level

Other, please state

4.5 **Please add any further comments that you believe are relevant**

Thank you for your time completing this survey.

**Dissemination workshop planned Spring 2021**
We will present initial results of the survey in a virtual workshop planned for May-June 2021. The aim of the workshop will be to offer information on TISP processes for HTA in low- and middle- income countries. The workshop will be open for all that have been invited to participate in the survey. An invitation will follow in due time.

If you have any questions, please email Elizabeth.Peacocke@fhi.no
