# Supplementary material for: Topic identification, selection, and prioritization for health technology assessment in selected countries: a mixed study design
Source: Cost Eff Resour Alloc. 2024 Feb 6;22:12. doi: 10.1186/s12962-024-00513-8 (PMC10848436; doi:10.1186/s12962-024-00513-8)
Supplement: Supplementary file 5 — Additional file 5: S5. Countries per region meeting the inclusion criteria. [file 12962_2024_513_MOESM5_ESM.docx]

**Supplementary File 5. Countries per region meeting the inclusion criteria**

We present sixty-four countries identified, and twenty-nine countries included for the survey sorted by regions. The World Bank classification (<https://datahelpdesk.worldbank.org/knowledgebase/articles/906519-world-bank-country-and-lending-groups>) and INAHTA membership when available is stated for those countries included in the survey. Below the table, we present the references supporting the selection provided.

**Table S4.1** Country list and classification

| Sixty-four countries identified with information on HTA | Twenty-nine countries included for the survey as potentially having a formalized HTA system (INAHTA member), World Bank classification and INAHTA membership when available. |
| --- | --- |
| Africa | |
| 1. Egypt 2. Ethiopia 3. Ghana 4. Iran 5. Jordan 6. Kuwait 7. Morocco 8. Malawi 9. Lebanon 10. Nigeria 11. Qatar 12. Saudi Arabia 13. South Africa 14. Sudan 15. Tanzania 16. Tunisia | 1. South Africa – Upper middle income 2. Iran- Islamic Rep – Lower middle income 3. Tunisia ([INEAS– National Authority for Assessment and Accreditation in Healthcare, TUNISIA](https://www.inahta.org/members/inasante/)) – Lower middle income |
| Asia | |
| 1. Bhutan 2. China 3. Hong Kong, China 4. India 5. Indonesia 6. Japan 7. Malaysia 8. Myanmar 9. Nepal 10. Philippines 11. Singapore 12. South Korea 13. Taiwan, China 14. Thailand 15. Vietnam | 1. Malaysia, Upper middle income 2. Singapore ([ACE– Agency for Care Effectiveness, SINGAPORE)](https://www.inahta.org/members/ace/) – High income 3. South Korea ([NECA– National Evidence-based healthcare Collaborating Agency, KOREA)](https://www.inahta.org/members/neca/) – High income 4. Thailand – Upper middle income 5. Taiwan ([CDE– Center for Drug Evaluation, Taiwan, REPUBLIC OF CHINA](https://www.inahta.org/members/cde/)) |
| Eastern Europe | |
| 1. Albania 2. Croatia 3. Bosnia 4. Bulgaria 5. Czech Republic 6. Bosnia Hercegovina 7. Hungary 8. Estonia 9. Greece 10. Kosovo 11. Khazakstan 12. Latvia 13. Lithuania 14. North Macedonia 15. Montenegro 16. Poland 17. Russia 18. Serbia 19. Slovakia 20. Slovenia 21. Turkey 22. Ukraine | 1. Bulgaria – Upper middle income 2. Croatia – High income 3. Czech Republic – High income 4. Estonia – High income 5. Hungary – High income 6. Kazakhstan ([RCHD – Ministry of Public Health of the Republic of Kazakhstan, Republican Centre for Health Development, KAZAKHSTAN)](https://www.inahta.org/members/rchd-cs/) – Upper middle 7. Latvia – High income 8. Lithuania – High income 9. Poland ([AOTMiT – Agency for Health Technology Assessment and Tariff System, POLAND](https://www.inahta.org/members/aotmit/) ) – High income 10. Romania – Upper middle income 11. Serbia – Upper middle income 12. Slovakia – High income 13. Turkey – Upper middle income 14. Ukraine ([SEC – Department of HTA at the State Expert Centre of the Ministry of Health, UKRAINE](https://www.inahta.org/members/sec/) ) – Lower middle income |
| Latin America | |
| 1. Argentina 2. Brazil 3. Chile 4. Colombia 5. Costa Rica 6. Ecuador 7. El Salvador 8. Mexico 9. Panama 10. Peru 11. Uruguay | 1. Argentina ([IECS – Institute for Clinical Effectiveness and Health Policy, ARGENTINA](https://www.inahta.org/members/iecs/) ) – Upper middle income 2. Brazil ([CONITEC – National Committee for Technology Incorporation, BRAZIL](https://www.inahta.org/members/conitec/) ) – Upper middle income 3. Chile – High income 4. Colombia – Upper middle income ([IETS – Instituto de Evaluación Tecnológica en Salud, COLOMBIA](https://www.inahta.org/members/iets/) ) 5. Mexico – Upper middle income 6. Uruguay ([AD-Uruguay – Health Assessment Division, Ministry of Public Health, URUGUAY)](https://www.inahta.org/members/msp/) – High income 7. Peru (IETSI – Institute of Health Technology Assessment and Research, PERU) – Upper middle income |

INAHTA= The International Network of Agencies for Health Technology Assessment, HTA = Health Technology Assessment

**References supporting evidence above**

**Africa:**

1. Addo, R., Hall, J., Haas, M., & Goodall, S. (2020). The knowledge and attitude of Ghanaian decision-makers and researchers towards health technology assessment. Social Science and Medicine, 250, 112889
2. Al-Aqeel, S. (2018). Health technology assessment in Saudi Arabia. Expert Review of Pharmacoeconomics & Outcomes Research, 18(4), 393-402.
3. Arab-Zozani, M., Sokhanvar, M., Kakemam, E., Didehban, T., & Hassanipour, S. (2020). History of Health Technology Assessment in Iran. International Journal of Technology Assessment in Health Care, 36(1), 34-39.
4. Darawsheh, B., & Germeni, E. (2019). Implementing health technology assessment in Kuwait: a qualitative study of perceived barriers and facilitators. International Journal of Technology Assessment in Health Care, 35(6), 422-426.
5. Doherty, J. E., Wilkinson, T., Edoka, I., & Hofman, K. (2017). Strengthening expertise for health technology assessment and priority-setting in Africa. Glob Health Action, 10(1), 1370194.
6. Fasseeh A, Karam R, Jameleddine M, et al. Implementation of Health Technology Assessment in the Middle East and North Africa: Comparison Between the Current and Preferred Status. Front Pharmacol. 2020;11:15. Published 2020 Feb 21. doi:10.3389/fphar.2020.00015
7. Hollingworth, S. A., Ruiz, F., Gad, M., & Chalkidou, K. (2020). Health technology assessment capacity at national level in sub-Saharan Africa: an initial survey of stakeholders. F1000Res, 9, 364.
8. Hollingworth, S., Gyansa-Lutterodt, M., Dsane-Selby, L., Nonvignon, J., Lopert, R., Gad, M., et al. (2020). Implementing health technology assessment in Ghana to support universal health coverage: building relationships that focus on people, policy, and process. International Journal of Technology Assessment in Health Care, 36(1), 8-11.
9. Hollingworth, S. A., Downey, L., Ruiz, FJ., Odame, E., Dsane-Selby, L., Gyansa-Lutterodt, et al. (2020). What do we need to know? Data sources to support evidence-based decisions using health technology assessment in Ghana. Health Research Policy and Systems, 18(1), 41.
10. Marsh, S. E., & Truter, I. (2020). The South African Guidelines for Pharmacoeconomic Submissions' Evidence Requirements Compared with Other African Countries and The National Institute for Health and Care Excellence in England. Expert Review of Pharmacoeconomics & Outcomes Research, 20(2), 155-168.
11. Odoch WD, Dambisya Y, Peacocke E, Sandberg KI, Hembre BSH. The role of government agencies and other actors in influencing access to medicines in three East African countries. Health Policy Plan 2021. 10.1093/heapol/czaa189
12. Panfilova, H., Nemchenko, O., Simonian, L., Tsurikova, O., & Bogdan, N. (2018). A comparative analysis of the willingness-to-pay indicators for the use of the innovative health technologies in Jordan, Egypt, Morocco, Sudan and Ethiopia. Journal of Pharmaceutical Sciences and Research, 10(8), 2097-2101
13. Surgey, G., Chalkidou, K., Reuben, W., Suleman, F., Miot, J., & Hofman, K. (2020). Introducing health technology assessment in Tanzania. International Journal of Technology Assessmentin Health Care, 36(2), 80-86.
14. Uzochukwu, B. S. C., Okeke, C., O'Brien, N., Ruiz, F., Sombie, I., & Hollingworth, S. (2020). Health technology assessment and priority setting for universal health coverage: a qualitative study of stakeholders' capacity, needs, policy areas of demand and perspectives in Nigeria. Global Health, 16(1), 58.
15. Zegeye, E. A., Reshad, A., Bekele, EA., Aurgessa, B., & Gella, Z. (2018). The State of Health Technology Assessment in the Ethiopian Health Sector: Learning from Recent Policy Initiatives. Value Health Reg Issues, 16, 61-65.

**Asia**

1. 1-Bae, E. Y. (2019). Role of Health Technology Assessment in Drug Policies: Korea. Value Health Reg Issues, 18, 24-29.
2. Chen, Y., Chi, X., He, Y., Wei, Y., Oortwijn, W., & Shi, L. (2019). Mapping of Health Technology Assessment in China: Situation Analysis and International Comparison. International Journal of Technology Assessment in Health Care, 35(5), 401-407.
3. Dabak, S. V., Teerawattananon, Y., & Win, T. (2019). From Design to Evaluation: Applications of Health Technology Assessment in Myanmar and Lessons for Low or Lower Middle-Income Countries. International Journal of Technology Assessment in Health Care, 35(6), 461-466.
4. Finkelstein EA, Krishnan A, Doble B. Beyond cost-effectiveness: A five-step framework for appraising the value of health technologies in Asia-Pacific. Int J Health Plann Manage. 2020;35(1):397-408.
5. Khowaja, A. R., Mitton, C., Qureshi, R., Bryan, S., Magee, L. A., von Dadelszen, P., & Bhutta, Z. A. (2017). Societal perspective on cost drivers for health technology assessment in Sindh, Pakistan. International Journal of Technology Assessment in Health Care, 33(2), 192-198.
6. Leelahavarong, P., Doungthipsirikul, S., Kumluang, S., Poonchai, A., Kittiratchakool, N., Chinnacom, D.,et al. (2019). Health Technology Assessment in Thailand: Institutionalization and Contribution to Healthcare Decision Making: Review of Literature. International Journal of Technology Assessment in Health Care, 35(6), 467-473.
7. Liu, G., Wu, E. Q., Ahn, J., Kamae, I., Xie, J., & Yang, H. (2020). The Development of Health Technology Assessment in Asia: Current Status and Future Trends. Value Health Reg Issues, 21, 39-44.
8. MacQuilkan, K., Baker, P., Downey, L., Ruiz, F., Chalkidou, K., Prinja, S.,et al. (2018). Strengthening health technology assessment systems in the global south: a comparative analysis of the HTA journeys of China, India and South Africa. Glob Health Action, 11(1), 1527556.
9. Prinja S, Rajsekhar K, Gauba VK. Health technology assessment in India: Reflection & future roadmap. Indian J Med Res 2020;152(5):444-7. 10.4103/ijmr.IJMR_115_19
10. Sharma M, Teerawattananon Y, Dabak SV, Isaranuwatchai W, Pearce F, Pilasant S, et al. A landscape analysis of health technology assessment capacity in the Association of South-East Asian Nations region. Health Res Policy Syst 2021;19(1):19. 10.1186/s12961-020-00647-0
11. Sharma, M., Teerawattananon, Y., Luz, A., Li, R., Rattanavipapong, W., & Dabak, S. (2020). Institutionalizing Evidence-Informed Priority Setting for Universal Health Coverage: Lessons From Indonesia. Inquiry, 57, 46958020924920.
12. Swami, S., & Srivastava, T. (2020). Role of Culture, Values, and Politics in the Implementation of Health Technology Assessment in India: A Commentary. Value in Health, 23(1), 39-42.
13. Singh, D., Luz, A. C. G., Rattanavipapong, W., & Teerawattananon, Y. (2017). Designing the Free Drugs List in Nepal: A Balancing Act Between Technical Strengths and Policy Processes. MDM Policy Pract, 2(1), 2381468317691766.
14. Takashi, F. (2018). A Pilot Program of Implementing Health Technology Assessment to Decision Making in Japan. Japanese Journal of Pharmacoepidemiology, 3-10.
15. Tanvejsilp, P., Taychakhoonavudh, S., Chaikledkaew, U., Chaiyakunapruk, N., & Ngorsuraches, S. (2019). Revisiting Roles of Health Technology Assessment on Drug Policy in Universal Health Coverage in Thailand: Where Are We? And What Is Next? Value Health Reg Issues, 18, 78-82.
16. Teerawattananon Y, Rattanavipapong W, Lin LW, Dabak SV, Gibbons B, Isaranuwatchai W, et al. Landscape analysis of health technology assessment (HTA): systems and practices in Asia. Int J Technol Assess Health Care. 2019;35(6):416-21
17. Vo, T. Q., & Pham, T. T. H. (2018). Health technology assessment in developing countries: A brief introduction for Vietnamese health-care policymakers. Asian Journal of Pharmaceutics, 12(1), S1-S7.
18. Wasir, R., Irawati, S., Makady, A., Postma, M., Goettsch, W., Feenstra, T., & Buskens, E. (2019). The implementation of HTA in medicine pricing and reimbursement policies in Indonesia: Insights from multiple stakeholders. PloS One, 14(11), e0225626.
19. Wong, C. K. H., Wu, O., & Cheung, B. M. Y. (2018). Towards a Transparent, Credible, Evidence-Based Decision-Making Process of New Drug Listing on the Hong Kong Hospital Authority Drug Formulary: Challenges and Suggestions. Appl Health Econ Health Policy, 16(1), 5-14.

**Eastern Europe**

1. Athanasakis, K. Thireos, E., Geitona, M., Yfantopoulos, J., & Kyriopoulos, J. (2020). A proposal for the procedures and organization of health technology assessment in Greece. Archives of Hellenic Medicine, 37(4), 439-444. 2)
2. Benisheva-Dimitrova, T., Sidjimova, D., Cherneva, D., & Kralimarkov, N. (2017). Pricing, reimbursement, and Health Technology Assessment of Medicinal products in Bulgaria. International Journal of Technology Assessment in Health Care, 33(3), 365-370.
3. Bucek Psenkova, M., Visnansky, M., Mackovicova, S., & Tomek, D. (2017). Drug Policy in Slovakia. Value Health Reg Issues, 13, 44-49.
4. -Csanádi, M., Inotai, A., Oleshchuk, O., Lebega, O., Alexandra, B., Piniazhko, O., et al. (2019). Health Technology Assessment Implementation in Ukraine: Current Status and Future Perspectives. International Journal of Technology Assessment in Health Care, 35(5), 393-400.
5. Csanádi, M., Ozierański, P., Löblová, O., King, L., Kaló, Z., & Botz, L. (2019). Shedding light on the HTA consultancy market: Insights from Poland. Health Policy, 123(12), 1237-1243.
6. Csanádi, M., Löblová, O., Ozierański, P., Harsányi, A., Kaló, Z., McKee, M., & King, L. (2019). When health technology assessment is confidential and experts have no power: the case of Hungary. Health Econ Policy Law, 14(2), 162-181.
7. Current status of health intervention and technology assessment in the Balkan region. Copenhagen: WHO Regional Office for Europe; 2020. Licence: CC BY-NC-SA 3.0 IGO.
8. García-Mochón, L., Espín Balbino, J., Olry de Labry Lima, A., Caro Martinez, A., Martin Ruiz, E., & Pérez Velasco, R. (2019). HTA and decision-making processes in Central, Eastern and South Eastern Europe: Results from a survey. Health Policy, 123(2), 182-190.
9. Khabibullina, A., & Gerry, C. J. (2019). Valuing Health States in Russia: A First Feasibility Study. Value Health Reg Issues, 19, 75-80.
10. Kahveci, R., Koç, E. M., & Küçük, E. (2017). Health technology assessment in Turkey. International Journal of Technology Assessment in Health Care, 33(3), 402-408.
11. Kosherbayeva, L., Hailey, D., Kurakbaev, K., Tabarov, A., Kumar, A., Gutzskaya, G., & Stepkina, E. (2016). A process of prioritizing topics for health technology assessment in Kazakhstan. International Journal of Technology Assessment in Health Care, 32(3), 147-151.
12. Lach, K., Dziwisz, M., Rémuzat, C., & Toumi, M. (2017). Towards a more transparent HTA process in Poland: new Polish HTA methodological guidelines. J Mark Access Health Policy, 5(1), 1355202.
13. Lipska, I., McAuslane, N., Leufkens, H., & Hövels, A. (2017). A decade of health technology assessment in Poland. International Journal of Technology Assessment in Health Care, 33(3), 350-357.
14. Mägi, K., Lepaste, M., & Szkultecka-Dębek, M. (2018). Drug Policy in Estonia. Value Health Reg Issues, 16, 1-4.
15. Németh, B., Csanádi, M., & Kaló, Z. (2017). Overview on the current implementation of the health technology assessment in the healthcare system in Hungary. International Journal of Technology Assessment in Health Care, 33(3), 333-338.
16. Ozturk, K., Karadayı, B., & Şener, O. (2018). Stakeholders’ perceptions of health technology assessment in Turkey. International Journal of Technology Assessment in Health Care, 34(1), 97-104
17. Rais, C., Kaló, Z., Csanádi, M., & Negulescu, V. (2020). Current and future perspectives for the implementation of health technology assessment in Romania. Health Policy and Technology, 9(1), 45-52.
18. Tsakalogiannis, C., Karampli, E., Athanasakis, K., & Kyriopoulos, J. (2019). The role of Health Technology Assessment in pharmaceutical policy decision-making in Greece. Findings from a qualitative study. Journal of Pharmaceutical Health Services Research, 10(4), 439-441.
19. Vassileva, M., Kamusheva, M., Manova, M., Savova, A., Tachkov, K., & Petrova, G. (2019). Historical overview of regulatory framework development on pricing and reimbursement of medicines in Bulgaria. Expert Review of Pharmacoeconomics & Outcomes Research, 19(6), 733-742.

**Latin America**

1. Armijos, L., Escalante, S., & Villacrés, T. (2017). [Health technology assessment in Ecuador's ministry of public health as a tool for drug purchasing from 2012 to 2015]. Revista Panamericana de Salud Publica, 41, e50. (Several general articles on HTA and LA from 2017 provide information)
2. Freiberg, A., Lafferriere, J. N., & Zambrano, M. (2019). Agnet y Judicialization en Salud en Argentina. Value Health Reg Issues, 20, 36-40.
3. Gilardino, R. E., Mejía, A., Guarín, D., Rey-Ares, L., & Perez, A. (2020). Implementing Health Technology Assessments in Latin America: Looking at the Past, Mirroring the Future. A Perspective from the ISPOR Health Technology Assessment Roundtable in Latin America. Value Health Reg Issues, 23, 6-12.
4. Gomes, P. T. C., Mata, V. E., Borges, T. C., & Galato, D. (2019). Horizon scanning in Brazil: outputs and repercussions. Revista de Saúde Publica, 53, 111.
5. Lavín, C. P., Alaniz, R., & Espinoza, M. (2017). Visions of Stakeholders about institutionalization of health technology assessment in Chile: A qualitative Study. International Journal of Technology Assessment in Health Care, 33(2), 303-306.
6. Lima, SGG., Brito, C, & Andrade, CJ (2019). O processo de incorporação de tecnologias em saúde no Brasil em uma perspectiva internacional. Ciênc. Saúde C-olet, 24(5), 1709-1722
7. Pichon-Riviere, A., Augustovski, F., García Martí, S., Alfie, V., & Sampietro-Colom, L. (2020). The link between health technology assessment and decision making for the allocation of health resources in Latin America. International Journal of Technology Assessment in Health Care, 36(2), 173-178.
