# Supplementary material for: Topic identification, selection, and prioritization for health technology assessment in selected countries: a mixed study design
Source: Cost Eff Resour Alloc. 2024 Feb 6;22:12. doi: 10.1186/s12962-024-00513-8 (PMC10848436; doi:10.1186/s12962-024-00513-8)
Supplement: Supplementary file 6 — Additional file 6: S6. Included country list by region. [file 12962_2024_513_MOESM6_ESM.pdf]

Region	Country identified	World Bank Clasification	HTA implementation status	Most recent relevant citation	INAHTA member organisation
Africa Sub-Sahara	Sub Sahara	LI-UMI	**Formalized:** South Africa<br>**Emerging**: other countries early phase	1) Hollingworth, S. A., Ruiz, F., Gad, M., & Chalkidou, K. (2020). Health technology assessment capacity at national level in sub-Saharan Africa: an initial survey of stakeholders. F1000Research, 9.<br>2) Doherty, J. E., Wilkinson, T., Edoka, I., & Hofman, K. (2017). Strengthening expertise for health technology assessment and priority-setting in Africa. Global health action, 10(1), 1370194.<br>3) Odoch, W. D., Dambisya, Y., Peacocke, E., Sandberg, K. I., & Hembre, B. S. H. (2021). The role of government agencies and other actors in influencing access to medicines in three East African countries. Health Policy and Planning, 36(3), 312-321.	None of the countries
Africa and Middle East	North (see individual countries Middle East)	LI-HI	**Formalized:** Saudi Arabia, Tunisia<br>**Emerging:** Egypt, (not mentioned in paper Iran, Kuwait)	1) Fasseeh, A., et al (2020). Implementation of health technology assessment in the Middle East and North Africa: comparison between the current and preferred status. Frontiers in pharmacology, 11, 15. 10.3389/fphar.2020.00015	No

Region	Country identified	World Bank Clasification	HTA implementation status	Most recent relevant citation	INAHTA member organisation
Africa Sub-Sahara	Ghana	LMI	**Emerging**	1) Hollingworth, S.A., et al. (2020). Implementing health technology assessment in Ghana to support universal health coverage: building relationships that focus on people, policy, and process. International Journal of Technology Assessment in Health Care, 36(1), 8-11.<br>2) Addo, R., Hall, J., Haas, M., & Goodall, S. (2020). The knowledge and attitude of Ghanaian decision-makers and researchers towards health technology assessment. Social Science & Medicine, 250, 112889.<br>3) Hollingworth, S. A. et al (2020). What do we need to know? Data sources to support evidence-based decisions using health technology assessment in Ghana. Health research policy and systems, 18(1), 1-12.	No
Africa Sub-Sahara	Malawi	LI	**Emerging**	1) Doherty, J. E., Wilkinson, T., Edoka, I., & Hofman, K. (2017). Strengthening expertise for health technology assessment and priority-setting in Africa. Global health action, 10(1), 1370194.	No
Africa Sub-Sahara	Nigeria	LMI	**Emerging**	1) Uzochukwu, B. S., Okeke, C., O'Brien, N., Ruiz, F., Sombie, I., & Hollingworth, S. (2020). Health technology assessment and priority setting for universal health coverage: a qualitative study of stakeholders' capacity, needs, policy areas of demand and perspectives in Nigeria. Globalization and health, 16(1), 1-11.	

Region	Country identified	World Bank Clasification	HTA implementation status	Most recent relevant citation	INAHTA member organisation
Africa Sub-Sahara	South Africa	UMI	**Formalized**	1)Marsh, S. E., & Truter, I. (2020). The South African Guidelines for Pharmacoeconomic Submissions' Evidence Requirements Compared with Other African Countries and The National Institute for Health and Care Excellence in England. Expert Review of Pharmacoeconomics & Outcomes Research, 20(2), 155-168.	No
Africa Sub-Sahara	Tanzania	LMI	**Emerging**	1) Surgey, G., Chalkidou, K., Reuben, W., Suleman, F., Miot, J., & Hofman, K. (2020). Introducing health technology assessment in Tanzania. International journal of technology assessment in health care, 36(2), 80-86.	No
Africa Sub-Sahara	Sudan	LI	**No information**	No information found, but mentioned in: Panfilova, H., Nemchenko, O., Simonian, L., Tsurikova, O., & Bogdan, N. (2018). A comparative analysis of the willingness-to-pay indicators for the use of the innovative health technologies in Jordan, Egypt, Morocco, Sudan and Ethiopia. Journal of Pharmaceutical Sciences and Research, 10(8), 2097-2101.	No
Africa Sub-Sahara	Ethiopia	LI	**Emerging**	1) Zegeye, E. A., Reshad, A., Bekele, E. A., Aurgessa, B., & Gella, Z. (2018). The state of health technology assessment in the Ethiopian health sector: learning from recent policy initiatives. Value in Health Regional Issues, 16, 61-65.	No
Africa and Middle East	Kuwait	HI	**Emerging**	1) Darawsheh, B., & Germeni, E. (2019). Implementing health technology assessment in Kuwait: a qualitative study of perceived barriers and facilitators. International Journal of Technology Assessment in Health Care, 35(6), 422-426.	No

Region	Country identified	World Bank Clasification	HTA implementation status	Most recent relevant citation	INAHTA member organisation
Africa and Middle East	Lebanon	UMI	**Emerging**	1) Abdel Rida, N., Mohamed Ibrahim, M. I., & Babar, Z. U. D. (2019). Pharmaceutical pricing policies in Qatar and Lebanon: narrative review and document analysis. Journal of Pharmaceutical Health Services Research, 10(3), 277-287. doi:10.3389/fphar.2020.00015;<br>2) Abdel Rida, N., Mohamed Ibrahim, M. I., & Babar, Z. U. D. (2019). Pharmaceutical pricing policies in Qatar and Lebanon: narrative review and document analysis. Journal of Pharmaceutical Health Services Research, 10(3), 277-287.	No
Africa and Middle East	Morocco	LMI	**No information**	1) Fasseeh A, Karam R, Jameleddine M, et al. Implementation of Health Technology Assessment in the Middle East and North Africa: Comparison Between the Current and Preferred Status. Front Pharmacol. 2020;11:15. Published 2020 Feb 21. doi:10.3389/fphar.2020.00015;<br>2) Panfilova, H., Nemchenko, O., Simonian, L., Tsurikova, O., & Bogdan, N. (2018). A comparative analysis of the willingness-to-pay indicators for the use of the innovative health technologies in Jordan, Egypt, Morocco, Sudan and Ethiopia. Journal of Pharmaceutical Sciences and Research, 10(8), 2097-2101.	No
Africa and Middle East	Qatar	HI	**Emerging:** HTA may be used in some contexts	1) Abdel Rida, N., Mohamed Ibrahim, M. I., & Babar, Z. U. D. (2019). Pharmaceutical pricing policies in Qatar and Lebanon: narrative review and document analysis. Journal of Pharmaceutical Health Services Research, 10(3), 277-287.	No

Region	Country identified	World Bank Clasification	HTA implementation status	Most recent relevant citation	INAHTA member organisation
Africa and Middle East	Saudi Arabia	HI	**Formalized** for high cost drugs	1) Fasseeh A, Karam R, Jameleddine M, et al. Implementation of Health Technology Assessment in the Middle East and North Africa: Comparison Between the Current and Preferred Status. Front Pharmacol. 2020;11:15. Published 2020 Feb 21. doi:10.3389/fphar.2020.00015;<br>2) Al-Aqeel, S. (2018). Health technology assessment in Saudi Arabia. Expert Review of Pharmacoeconomics & Outcomes Research, 18(4), 393-402.	No
Africa and Middle East	Iran, Islamic Rep	UMI	**Formalized**	1) Arab-Zozani, M., Sokhanvar, M., Kakemam, E., Didehban, T., & Hassanipour, S. (2020). History of Health Technology Assessment in Iran. International Journal of Technology Assessment in Health Care, 36(1), 34-39.	No
Africa and Middle East	Jordan	UMI	**Emerging**	1) Mentioned in: Panfilova, H., Nemchenko, O., Simonian, L., Tsurikova, O., & Bogdan, N. (2018). A comparative analysis of the willingness-to-pay indicators for the use of the innovative health technologies in Jordan, Egypt, Morocco, Sudan and Ethiopia. Journal of Pharmaceutical Sciences and Research, 10(8), 2097-2101.	No

Region	Country identified	World Bank Clasification	HTA implementation status	Most recent relevant citation	INAHTA member organisation
Africa and Middle East	Egypt	LMI	**Emerging**	1) Fasseeh A, Karam R, Jameleddine M, et al. Implementation of Health Technology Assessment in the Middle East and North Africa: Comparison Between the Current and Preferred Status. Front Pharmacol. 2020;11:15. Published 2020 Feb 21. doi:10.3389/fphar.2020.00015;<br>2) mentioned in: Panfilova, H., Nemchenko, O., Simonian, L., Tsurikova, O., & Bogdan, N. (2018). A comparative analysis of the willingness-to-pay indicators for the use of the innovative health technologies in Jordan, Egypt, Morocc	Yes - Central Administration of Health Technology Assessment, Egyptian Authority for unified Procurement, Medical Supplies and Technology Management Agency
Africa and Middle East	Tunisia	LMI	**Formalized** (little information)	Fasseeh A, Karam R, Jameleddine M, et al. Implementation of Health Technology Assessment in the Middle East and North Africa: Comparison Between the Current and Preferred Status. Front Pharmacol. 2020;11:15. Published 2020 Feb 21. doi:10.3389/fphar.2020.00015;	INEAS – National Authority for Assessment and Accreditation in Healthcare, TUNISIA

Region	Country identified	World Bank Clasification	HTA implementation status	Most recent relevant citation	INAHTA member organisation
**Latin America**	Latin America	UMI-HI	**Formalized** HTA systems in: Argentina; Brazil; Chile; Colombia; Peru; Uruguay; Ecuador; Colombia; Costa Rica; El Salvador; Mexico; Panama; Peru.	1) Gilardino, R. E., Mejía, A., Guarín, D., Rey-Ares, L., & Perez, A. (2020). Implementing Health Technology Assessments in Latin America: Looking at the Past, Mirroring the Future. A Perspective from the ISPOR Health Technology Assessment Roundtable in Latin America. Value Health Reg Issues, 23, 6-12.; 2) Pichon-Riviere, A., Augustovski, F., García Martí, S., Alfie, V., & Sampietro-Colom, L. (2020). The link between health technology assessment and decision making for the allocation of health resources in Latin America. International Journal of Technology Assessment in Health Care, 36(2), 173-178.	See below
Latin America	Argentina	UMI (june 2020)	Established agency, not formalized	1) Freiberg, A., Lafferriere, J. N., & Zambrano, M. (2019). AGNET and Judicialization in Health in Argentina. Value in Health Regional Issues, 20, 36-40.	IECS – Institute for Clinical Effectiveness and Health Policy, ARGENTINA
Latin America	Brazil	UMI	**Formalized**	1) Lima, S. G. G., Brito, C. d., & Andrade, C. J. C. d. (2019). O processo de incorporação de tecnologias em saúde no Brasil em uma perspectiva internacional. Ciênc. Saúde Colet, 24(5), 1709-1722. 2) Gomes, P. T. C., Mata, V. E., Borges, T. C., & Galato, D. (2019). Horizon scanning in Brazil: outputs and repercussions. Revista de Saúde Publica, 53, 111.	CONITEC – National Committee for Technology Incorporation, BRAZIL

Region	Country identified	World Bank Clasification	HTA implementation status	Most recent relevant citation	INAHTA member organisation
Latin America	Chile	HI	**Formalized**	1) Lavín, C. P., Alaniz, R., & Espinoza, M. (2017). Visions of stakeholders about institutionalization of health technology assessment in Chile: a qualitative study. International Journal of Technology Assessment in Health Care, 33(2), 303-306.	No
Latin America	Colombia	UMI	**Formalized**	1) Castro, H. E., Moreno-Mattar, O., & Rivillas, J. C. (2018). HTA and MCDA solely or combined? The case of priority-setting in Colombia. Cost Eff Resour Alloc, 16(Suppl 1), 47.	IETS – Instituto de Evaluación Tecnológica en Salud, COLOMBIA
Latin America	Ecuador	UMI	HTA implemented, but unclear how formalized (limited information)	1) Armijos, L., Escalante, S., & Villacrés, T. (2017). [Health technology assessment in Ecuador's ministry of public health as a tool for drug purchasing from 2012 to 2015]. Revista Panamericana de Salud Publica, 41, e50. (Several general articles on HTA and LA from 2017 provide information)	No
Latin America	Mexico	UMI	**Formalized** (limited information)	Information in the general citations from Latin America	No
Latin America	Uruguay	HI	**Formalized** (limited information)	Information in the general citations from Latin America	AD-Uruguay – Health Assessment Division, Ministry of Public Health, URUGUAY

Region	Country identified	World Bank Clasification	HTA implementation status	Most recent relevant citation	INAHTA member organisation
Latin America	Peru	UMI	**Formalized** (limited information)	Information in the general citations from Latin America	IETSI – Institute of Health Technology Assessment and Research, PERU

Region	Country identified	World Bank Clasification	HTA implementation status	Most recent relevant citation	INAHTA member organisation
Asia-Pacific	Asia-Pacific	LMI-HI	**Formalized** systems: Taiwan, South Korea, Thailand, Taiwan, Malaysia and Singapore ; HTA processes established or under establishment: Vietnam, Philippines, China, and India, Japan; (Hong Kong) Early phase: Nepal, Bhutan, Indonesia	1) Teerawattananon, Y., et al (2019). Landscape analysis of health technology assessment (HTA): systems and practices in Asia. International journal of technology assessment in health care, 35(6), 416-421;<br>2) Liu, G., Wu, E. Q., Ahn, J., Kamae, I., Xie, J., & Yang, H. (2020). The Development of Health Technology Assessment in Asia: Current Status and Future Trends. Value Health Reg Issues, 21, 39-44.;<br>3) Finkelstein, E. A., Krishnan, A., & Doble, B. (2020). Beyond cost-effectiveness: A five-step framework for appraising the value of health technologies in Asia-Pacific. The International Journal of Health Planning and Management, 35(1), 397-408.<br>4) MacQuilkan, K. et al. (2018). Strengthening health technology assessment systems in the global south: a comparative analysis of the HTA journeys of China, India and South Africa. Glob Health Action, 11(1), 1527556.<br>5) Sharma M, et al. (2021). A landscape analysis of health technology assessment capacity in the Association of South-East Asian Nations region. Health research policy and systems, 19, 1-13.	See below
Asia-Pacific	China	UMI	**Emerging** (a large amount of information on the ongoing process)	1) Chen, Y., Chi, X., He, Y., Wei, Y., Oortwijn, W., & Shi, L. (2019). Mapping of Health Technology Assessment in China: Situation Analysis and International Comparison. International Journal of Technology Assessment in Health Care, 35(5), 401-407.	No

Region	Country identified	World Bank Clasification	HTA implementation status	Most recent relevant citation	INAHTA member organisation
Asia-Pacific	Hong Kong, SAR, China	HI	**Emerging** (limited information)	1) Wong, C. K. H., et al. (2018). Towards a Transparent, Credible, Evidence-Based Decision-Making Process of New Drug Listing on the Hong Kong Hospital Authority Drug Formulary: Challenges and Suggestions. Appl Health Econ Health Policy, 16(1), 5-14.	No
Asia-Pacific	India	LMI	**Emerging**	1) Swami, S., & Srivastava, T. (2020). Role of Culture, Values, and Politics in the Implementation of Health Technology Assessment in India: A Commentary. Value in Health, 23(1), 39-42.;<br>2)Prinja, S., Rajsekhar, K., & Gauba, V. K. (2020). Health technology assessment in India: Reflection & future roadmap. The Indian Journal of Medical Research, 152(5), 444. 10.4103/ijmr.IJMR_115_19	No
Asia-Pacific	Indonesia	UMI	**Emerging**	1) Sharma, M., Teerawattananon, Y., Luz, A., Li, R., Rattanavipapong, W., & Dabak, S. (2020). Institutionalizing Evidence-Informed Priority Setting for Universal Health Coverage: Lessons From Indonesia. Inquiry, 57, 46958020924920.;<br>2) Wasir, R., Irawati, S., Makady, A., Postma, M., Goettsch, W., Feenstra, T., & Buskens, E. (2019). The implementation of HTA in medicine pricing and reimbursement policies in Indonesia: Insights from multiple stakeholders. PloS One, 14(11), e0225626.	
Asia-Pacific	Japan	HI	**Emerging**	1) Takashi, F. (2018). A Pilot Program of Implementing Health Technology Assessment to Decision Making in Japan. Japanese Journal of Pharmacoepidemiology, 3-10.	

Region	Country identified	World Bank Clasification	HTA implementation status	Most recent relevant citation	INAHTA member organisation
Asia-Pacific	Malaysia	UMI	**Formalized**	1) Roza, S., et al. (2019). Health Technology Assessment in Malaysia: Past, Present, and Future. International Journal of Technology Assessment in Health Care, 35(6), 446-451.	MaHTAS – Health Technology Assessment Section, Ministry of Health Malaysia, MALAYSIA
Asia-Pacific	Myanmar	LMI	Implemented in a program (vertical government program on maternal and child health care)	1) Dabak, S. V., Teerawattananon, Y., & Win, T. (2019). From Design to Evaluation: Applications of Health Technology Assessment in Myanmar and Lessons for Low or Lower Middle-Income Countries. International Journal of Technology Assessment in Health Care, 35(6), 461-466.	No
Asia-Pacific	Nepal	LMI	**Emerging**	1) Singh, D., Luz, A. C. G., Rattanavipapong, W., & Teerawattananon, Y. (2017). Designing the Free Drugs List in Nepal: A Balancing Act Between Technical Strengths and Policy Processes. MDM Policy Pract, 2(1), 2381468317691766.	No

Region	Country identified	World Bank Clasification	HTA implementation status	Most recent relevant citation	INAHTA member organisation
Asia-Pacific	Philippines	LMI	**Emerging**	1) Vlad, I. (2020). Establishing health technology assessment (HTA) in middle-income countries: a comparative analysis of the path towards institutionalisation in Thailand and the Philippines (Doctoral dissertation, London School of Hygiene & Tropical Medicine).<br>2) Uezono, D., Fajardo, M., Zuniga, Y. M., Briones, J., Genuino, A. J., Guerrero, A. M., & Castro, B. (2020). Review of Philippine Health Laws and its Implication to HTA Institutionalization. European Journal of Public Health, 30(Supplement_5), ckaa166-590.	No
Asia-Pacific	Pakistan	LMI	**Emerging**	1) Khowaja, A. R.,et al. (2017). Societal Perspective on cost drivers for health technology assessment in Sind, Pakistan. International Journal of Technology Assessment in Health Care, 33(2), 192-198.	No
Asia-Pacific	Singapore	HI	**Formalized**	1) Pearce, F., Lin, L., Teo, E., Ng, K., & Khoo, D. (2019). Health Technology Assessment and Its Use in Drug Policies: Singapore. Value Health Reg Issues, 18, 176-183.	ACE – Agency for Care Effectiveness, SINGAPORE
Asia-Pacific	Korea, Rep (South Korea)	HI	**Formalized**	1) Bae, E. Y. (2019). Role of Health Technology Assessment in Drug Policies: Korea. Value Health Reg Issues, 18, 24-29.;<br>2) Bae, E. Y., et al. (2018). Role of economic evidence in coverage decision-making in South Korea. PloS One, 13(10), e0206121.	NECA – National Evidence-based healthcare Collaborating Agency, KOREA

Region	Country identified	World Bank Clasification	HTA implementation status	Most recent relevant citation	INAHTA member organisation
Asia-Pacific	Taiwan	HI	**Formalized**	1) Kennedy-Martin, T. et al. (2014). The health technology assessment environment in mainland China, Japan, South Korea, and Taiwan—implications for the evaluation of diabetes mellitus therapies. Value in Health Regional Issues, 3, 108-116.<br>2)Kamae, I. (2010). Value-based approaches to healthcare systems and pharmacoeconomics requirements in Asia: South Korea, Taiwan, Thailand and Japan. Pharmacoeconomics, 28, 831-838.	CDE – Center for Drug Evaluation, Taiwan, REPUBLIC OF CHINA
Asia-Pacific	Thailand	UMI	**Formalized**	1) Leelahavarong, P. et al. (2019). Health Technology Assessment in Thailand: Institutionalization and Contribution to Healthcare Decision Making: Review of Literature. International Journal of Technology Assessment in Health Care, 35(6), 467-473.;<br>2) Tanvejsilp, P. et al. (2019). Revisiting Roles of Health Technology Assessment on Drug Policy in Universal Health Coverage in Thailand: Where Are We? And What Is Next? Value Health Reg Issues, 18, 78-82.	No
Asia-Pacific	Vietnam	LMI	**Emerging**	1) Vo, T. Q., & Pham, T. T. H. (2018). Health technology assessment in developing countries: A brief introduction for Vietnamese health-care policymakers. Asian Journal of Pharmaceutics, 12(1), S1-S7.	No

Region	Country identified	World Bank Clasification	HTA implementation status	Most recent relevant citation	INAHTA member organisation
Eastern Europe and Central Asia	Balkan region	UMI-HI (lower range of HI)	Not implemented or emerging: Only Serbia classifies as formalized system; but to some degree Croatia has an HTA system (reduced very much in 2018-	1) World Health Organization. (2020). Current status of health intervention and technology assessment in the Balkan region (No. WHO/EURO: 2020-1303-41053-55733). World Health Organization. Regional Office for Europe..	See below
Eastern Europe and Central Asia	Central Eastern Europe	UMI-HI (lower range of HI)	**Formalized** HTA: Bulgaria; Croatia; Czech Republic; Estonia; Greece; Hungary; Lithuania; Latvia; Poland; Republic of Serbia; Russia; Slovenia; Slovakia; Serbia.	1) García-Mochón, L., Espín Balbino, J., Olry de Labry Lima, A., Caro Martinez, A., Martin Ruiz, E., & Pérez Velasco, R. (2019). HTA and decision-making processes in Central, Eastern and South Eastern Europe: Results from a survey. Health Policy, 123(2), 182-190.<br>2) World Health Organization. (2020). Current status of health intervention and technology assessment in the Balkan region (No. WHO/EURO: 2020-1303-41053-55733). World Health Organization. Regional Office for Europe.	See below
Eastern Europe and Central Asia	Bosnia Hercegovina	UMI	**Emerging**	1) World Health Organization. (2020). Current status of health intervention and technology assessment in the Balkan region (No. WHO/EURO: 2020-1303-41053-55733). World Health Organization. Regional Office for Europe.z	No

Region	Country identified	World Bank Clasification	HTA implementation status	Most recent relevant citation	INAHTA member organisation
Eastern Europe and Central Asia	Bulgaria	UMI	**Formalized**	1) Vassileva, M., Kamusheva, M., Manova, M., Savova, A., Tachkov, K., & Petrova, G. (2019). Historical overview of regulatory framework development on pricing and reimbursement of medicines in Bulgaria. Expert Review of Pharmacoeconomics & Outcomes Research, 19(6), 733-742.;<br>2) Benisheva-Dimitrova, T., Sidjimova, D., Cherneva, D., & Kralimarkov, N. (2017). Pricing, reinbursement, and health technology assessment of medicinal products in Bulgaria. International Journal of Technology Assessment in Health Care, 33(3), 365-370.	No
Eastern Europe and Central Asia	Croatia	UMI	**Emerging**	1) World Health Organization. (2020). Current status of health intervention and technology assessment in the Balkan region (No. WHO/EURO: 2020-1303-41053-55733). World Health Organization. Regional Office for Europe.	No
Eastern Europe and Central Asia	Czech Republic	HI	**Formalized** (limited information)	1) García-Mochón, L., Espín Balbino, J., Olry de Labry Lima, A., Caro Martinez, A., Martin Ruiz, E., & Pérez Velasco, R. (2019). HTA and decision-making processes in Central, Eastern and South Eastern Europe: Results from a survey. Health Policy, 123(2), 182-190.	No
Eastern Europe and Central Asia	Estonia	HI	**Formalized**	1) Mägi, K., Lepaste, M., & Szkultecka-Dębek, M. (2018). Drug Policy in Estonia. Value Health Reg Issues, 16, 1-4.	No

Region	Country identified	World Bank Clasification	HTA implementation status	Most recent relevant citation	INAHTA member organisation
Eastern Europe and Central Asia	Greece	HI	**Emerging**	1) Athanasakis, K., Thireos, E., Geitona, M., Yfantopoulos, J., & Kyriopoulos, J. (2020). A proposal for the procedures and organization of health technology assessment in Greece. Archives of Hellenic Medicine, 37(4), 439-444.<br>2) Tsakalogiannis, C., Karampli, E., Athanasakis, K., & Kyriopoulos, J. (2019). The role of Health Technology Assessment in pharmaceutical policy decision-making in Greece. Findings from a qualitative study. Journal of Pharmaceutical Health Services Research, 10(4), 439-441.	No
Eastern Europe and Central Asia	Hungary	HI (lower range)	**Formalized**	1) Németh, B., Csanádi, M., & Kaló, Z. (2017). Overview on the current implementation of health technology assessment in the healthcare system in Hungary. International Journal of Technology Assessment in Health Care, 33(3), 333-338.;<br>2) Csanádi, M., Löblová, O., Ozierański, P., Harsányi, A., Kaló, Z., McKee, M., & King, L. (2019). When health technology assessment is confidential and experts have no power: the case of Hungary. Health Econ Policy Law, 14(2), 162-181.	RCHD – Ministry of Public Health of the Republic of Kazakhstan, Republican Centre for Health Development, KAZAKHSTAN
Eastern Europe and Central Asia	Kazakhstan	UMI	**Formalized**	1) Kosherbayeva, L., Hailey, D., Kurakbaev, K., Tabarov, A., Kumar, A., Gutzskaya, G., & Stepkina, E. (2016). A process of prioritizing topics for health technology assessment in Kazahstan. International Journal of Technology Assessment in Health Care, 32(3), 147-151.	No

**Region**	**Country identified**	**World Bank Clasification**	**HTA implementation status**	**Most recent relevant citation**	**INAHTA member organisation**
Eastern Europe and Central Asia	Latvia	HI	**Formalized** (limited information)	1) García-Mochón, L., Espín Balbino, J., Olry de Labry Lima, A., Caro Martinez, A., Martin Ruiz, E., & Pérez Velasco, R. (2019). HTA and decision-making processes in Central, Eastern and South Eastern Europe: Results from a survey. Health Policy, 123(2), 182-190.	No
Eastern Europe and Central Asia	Lithuania	HI	**Formalized**	1) García-Mochón, L., Espín Balbino, J., Olry de Labry Lima, A., Caro Martinez, A., Martin Ruiz, E., & Pérez Velasco, R. (2019). HTA and decision-making processes in Central, Eastern and South Eastern Europe: Results from a survey. Health Policy, 123(2), 182-190.	No
Eastern Europe and Central Asia	Poland	HI	**Formalized**	1) Lipska, I., McAuslane, N., Leufkens, H., & Hövels, A. (2017). A decade of health technology assessment in Poland. International Journal of Technology Assessment in Health Care, 33(3), 350-357.; 2) Csanádi, M., Ozierański, P., Löblová, O., King, L., Kaló, Z., & Botz, L. (2019). Shedding light on the HTA consultancy market: Insights from Poland. Health Policy, 123(12), 1237-1243.; 3) Lach, K., Dziwisz, M., Rémuzat, C., & Toumi, M. (2017). Towards a more transparent HTA process in Poland: new Polish HTA methodological guidelines. J Mark Access Health Policy, 5(1), 1355202.	AOTMiT – Agency for Health Technology Assessment and Tariff System, POLAND
Eastern Europe and Central Asia	Romania	HI	**Emerging**	1) Rais, C., Kaló, Z., Csanádi, M., & Negulescu, V. (2020). Current and future perspectives for the implementation of health technology assessment in Romania. Health Policy and Technology, 9(1), 45-52.	

Region	Country identified	World Bank Clasification	HTA implementation status	Most recent relevant citation	INAHTA member organisation
Eastern Europe and Central Asia	Russia	UMI	**Emerging**	1) Khabibullina, A., & Gerry, C. J. (2019). Valuing Health States in Russia: A First Feasibility Study. Value Health Reg Issues, 19, 75-80.	CHQAC – Center for Healthcare Quality Assessment and Control, RUSSIAN FEDERATION
Eastern Europe and Central Asia	Serbia	UMI	**Formalized**	1) World Health Organization. (2020). Current status of health intervention and technology assessment in the Balkan region (No. WHO/EURO: 2020-1303-41053-55733). World Health Organization. Regional Office for Europe.	No
