# Supplementary material for: Topic identification, selection, and prioritization for health technology assessment in selected countries: a mixed study design
Source: Cost Eff Resour Alloc. 2024 Feb 6;22:12. doi: 10.1186/s12962-024-00513-8 (PMC10848436; doi:10.1186/s12962-024-00513-8)
Supplement: Supplementary file 7 — Additional file 7: S7. Summary of findings. [file 12962_2024_513_MOESM7_ESM.pdf]

Survey on Topic Identification, Selection and Prioritisation (TISP) processes for Health Technology Assessment (HTA)

SUMMARY OF FINDINGS AND DISCUSSION

Why were you selected to participate: You were selected to participate in this survey as your country was identified in our literature review as an African, Asian, Latin American or Eastern European country with a formalised HTA system.

The aims of this survey were to:
- explore how Topic Identification, Selection and Prioritization (TISP) is performed in selected African, Asian, Latin American and European countries with a formalised HTA system
- seek information on what has influenced a country's choice of option for TISP
- seek information on what is considered as future needs for TISP in a country's HTA system(s)

MAIN RESULTS OF THE SURVEY

Results from the literature review:

- A systematic literature review identified 29 countries with a potential recently formalized Health Technology Assessment (HTA) system from Africa (2), Asia Pacific (5), Latin America (7), Europe (14), and the Middle East (1). Among these, 3 were middle-income, 13 were upper-middle-income, and 11 were high-income countries.
- A survey was conducted in 23 of the identified countries, with a 72% response rate.

Results from the survey:

- **HTA System background information:** The scope of technologies assessed includes medicines for specialized care, primary care, high-cost care, medical devices, and public health programmes. The largest volume of assessments is for pharmaceuticals, ranging from 21 to 50 products per year. Formal decision makers include policy makers, payers and insurance agencies, and committees, with variations between centralized and regional processes.
- **How TISP is performed**: The survey results on *identification approaches* showed significant differences. Out of the total respondents (n=22), around half (n=11) preferred a proactive approach to identify topics through a formalized process, while fewer participants (n=5) relied on horizon scanning or early warning systems. The most common approach was to have topic proposals suggested by government officials, departments, or HTA systems decision makers (n=16). In addition, almost all respondents selected that more than one stakeholder group was involved in the identification of topics (n=18), indicating that there is the framework of an integrated process in most of the participating countries. Topics were equally proposed by health care workers/experts (n=13) and manufacturers (n=13).
- The survey results showed mixed responses regarding the use of explicit criteria for topic *selection and prioritization*. Nine respondents reported using explicit processes and criteria for *prioritization*. Of those, six shared the criteria they used, including burden of disease, cost impact, clinical and organizational effects, and national ethical and equity considerations. One respondent used the Pritec tool, while two provided links to publicly available criteria. Three respondents used criteria but did not provide details, and one mentioned that criteria use was in a pilot phase.

- The availability and transparency of TISP outcomes also varies, with some respondents indicating that information is not publicly available, while others reported efforts to improve transparency.
- **Factors influencing the current TISP process:** Eleven respondents reported that the main factor influencing countries' current TISP processes was the role policy makers and expert involvement. Other factors that were reported to influence the TISP process include the existence of participatory processes involving all or most relevant stakeholders (n=eight), political decision (n=six), and influence of international or regional networks and collaborations on TISP processes (n=eight). The pharmaceutical industry or professional societies were also important, with this highlighted as an issue of concern in considerations related to high-cost oncological drugs
- **Future needs**: Respondents highlighted limitations in current TISP processes such as lack of topic selection processes, weak regulation, incomplete criteria, and decision being irrelevant for political decision-making. Challenges in terms of lack of human resource capacity and fragmentation of HTA as a decision-making tool in the health sector were also noted, e.g., that HTA may not be integrated into other health system decision-making processes. Some respondents reported attempts to improve TISP processes, such as adjusting the type or weighting criteria, improving transparency, and building capacity.

DISCUSSION

- This study investigated the range of approaches to technology identification and selection processes for HTA systems in selected countries. The study found variation in TISP approaches, with various factors influencing the approach including political influence, limited involvement of experts, and limited participatory processes involving relevant stakeholders. The study also highlighted the importance of political buy-in for successful HTA implementation, with perceptions of inequality and lack of confidence in the feasibility of the process being successful being potential barriers to achieving the full potential of TISP/HTA.
- This survey found that the degree of patient and citizen involvement in the TISP process for HTA is relatively low, with greater involvement from clinical experts, government policymakers, and private manufacturers. Increasing public involvement may improve transparency and evidence-based decision-making. Examples from other sectors highlight the effectiveness of stakeholder engagement in decision-making in terms of achieving programme success. However, this would lead to greater demands on human resources capacity and coordination skills to ensure all relevant representatives are engaged. Public awareness raising activities and formal feedback processes may increase the level of patients' and citizen groups' engagement in health policy decisions.
- Despite efforts to improve TISP processes, challenges remain. For example, participants indicated support for adapting TISP approaches for different contexts. Explicit TISP criteria and public availability of information on decisions can increase the transparency of the HTA and TISP process, especially in countries with several prioritization processes and multiple HTAs to consider. Improving TISP processes would require consideration of the overall HTA system, including an assessment of what additional resources are needed. Enshrining TISP and HTA in health care decision-making is crucial and legislation may be necessary to achieve this. Increased public engagement and ownership of health care decision making may generate more enthusiasm for priority-setting processes. Multi-stakeholder involvement in HTA, accompanied by clear processes for managing conflicts of interest, can support evidence-informed health policy.
