# Supplementary material for: Topic identification, selection, and prioritization for health technology assessment in selected countries: a mixed study design
Source: Cost Eff Resour Alloc. 2024 Feb 6;22:12. doi: 10.1186/s12962-024-00513-8 (PMC10848436; doi:10.1186/s12962-024-00513-8)
Supplement: Supplementary file 8 — Additional file 8: S8. Interview guide. [file 12962_2024_513_MOESM8_ESM.pdf]

Participant Information Sheet

Study title: *Survey on Topic Identification, Selection and Prioritisation (TISP) processes for Health Technology Assessment (HTA).*

Thank you for agreeing to participate in this follow up interview. Your contribution to this research is greatly appreciated, and we thank you for your time and expertise.

- Before we begin, please take a moment to review the *Summary of findings and discussion*.
- We are asking you to respond in your professional capacity and to draw upon your personal knowledge and experiences for this study.
- All data collected during this interview will be kept confidential, anonymised, and store for a maximum of 5 years in a password protected computer at the Norwegian Institute of Public Health.
- Your participation in this interview is voluntary. You are free to withdraw at any time and without prejudice.
- This study has obtained a waiver for *ethical clearance* from the Norwegian Ethics Regional Committees for Medical and Healthcare Professional (application #586503), on the basis that it is not concerned with health and illness, and therefore does not fall within the scope of the Health Research Act
- All participants are treated with confidentiality and that participants are free to decide if participation is right for them.
- If you have questions or require more information about the study, please contact Elizabeth Peacocke at elizabet.peacocke@fhi.no or Julia Bidonde Julia.bidonde@fhi.no

---

Interview Guide

Establishing questions

0. Your country – this interview content will be anonymised.
1. Do you consider yourself to have the necessary experience and understanding of the HTA system in the country to respond to questions about TISP processes? (from the original survey)

General questions

2. Do you have an example of a country or countries where you think the TISP process is particularly successful and/or well established?
    1. What are the features of the process in that country/countries that you think contribute to its success from your perspective?
    2. What could be improved in the TISP process in that country?
3. To what degree do the survey findings agree with your own experience of issues around the TISP process?
    1. Please elaborate.
4. Do you feel that there are issues that the survey has not highlighted, that are important to the uptake of TISP?
    1. Please elaborate.

Questions around the main points from the Discussion and Conclusions

5. "The main factors influencing the chosen approach to TISP....were that it is a process limited to policy makers and expert involvement, the second most common response was that it was a participatory process involving all or most relevant stakeholders. For a section of respondents, the political influence was an important factor, and was also mentioned in several open text responses. It was reported that there is both a real and perceived political influence on HTA decisions such as what topics to select."
    a. Probe about ways to move to a more participatory process - Please describe how your country supports inclusiveness of different stakeholders in the TISP process?
    b. Probe about impartiality
        1. How to cope with pressures from stakeholders to focus on specific topics? How to manage the process so that it is more objective?
        2. Are there any measures that your country takes to support impartiality, meaning that vested interests are objectively managed, in TISP?
6. The TISP process seems to require political "buy-in" to flourish. How can political "buy-in" be achieved based on your experience or opinion?
7. "In this survey, TISP was characterized as having relatively low patient and citizen involvement, but has had a significant contribution from clinical experts, government policymakers and private manufacturers."
    a. Based on your experience/opinion, why is there low patient and citizen involvement in some countries? What are the barriers/challenges?
    b. How do you think patient and citizen involvement in TISP could be increased? What successful methods are you aware of?
    c. What would be your criteria for assessing patient and citizen involvement in TISP as adequate?
8. In this survey "Despite countries working to improve their TISP processes by including more explicit criteria and making final decisions more accessible and publicly available, there continues to be challenges with the TISP methodology in terms of adaptation to different context and developing one standard methods guidance".
    a. Does this reflect your experience? Please elaborate.
    b. How do you think prioritization processes for question selection can be made more explicit? What has worked well from your perspective/experience? What barriers need to be overcome?
    c. How do you think final decisions can be made more accessible to the public?
9. The survey suggests that "... a primary policy implication of this study is the need for TISP and HTA to be enshrined within the fabric of health care decision-making and priority setting in countries".
    a. Do you think this assessment makes sense or are other approaches effective as well?
    b. Can you think of countries where this embedded approach has been achieved? How have these countries reached this situation e.g. Through legislation or other means?
10. The management of conflict of interest is seen as a key contributor to transparency in TISP.
    a. Do you agree with this statement? Please elaborate.
    b. Are you aware of good examples of COI processes which you feel have contributed to the development or uptake of TISP?
11. Resource questions
    a. Do you think that resources are a major issue hampering the uptake of TISP? Which resources specifically?
    b. In your experience have collaborative efforts within TISP been successful? Please elaborate?
